# Gap Flow Simulation Methods in High Pressure Variable Displacement Axial Piston Pumps

**DOI:** 10.1007/s11831-016-9180-5

**Published:** 2016-04-26

**Authors:** Tomasz Zawistowski, Michał Kleiber

**Affiliations:** 1 Space Research Centre, Polish Academy of Sciences, Warsaw, Poland; 2 Institute of Fundamental Technological Research, Polish Academy of Sciences, Warsaw, Poland

## Abstract

High pressure variable displacement axial piston pumps are subject to complex dynamic phenomena. Their analysis is difficult, additionally complicated by leakage of the working fluid. Analytically gap flow is calculated with the Reynolds equation which describes the pressure distribution in a thin lubricating layer. The paper presents various approaches to analyze gap flow both in traditional axial piston pump and novel type of hydraulic pump, designed at the Polish Gdansk Institute of Technology. Because of large aspect ratio between the height of the gap and the size of pump elements, the authors present the numerical simulation approach using a local model to define a lubrication gap, linked to a global model of a pump from which boundary conditions were imported. User defined functions implemented in Fluent and Excel were used to calculate the pressure and velocity fields and assess the fluid flow rate.

## Introduction

It is owing to advances in computation technology that numerical simulation of complicated dynamic phenomena became possible. Nevertheless not all details can be included in one model. One of the reasons could be a large aspect ratio between different parts of the object under study. In the case of hydraulic axial piston pumps a difference between the size of gap height and the displacement chamber diameter can reach three orders of magnitude and could render the analysis results unreliable, since they would be vitiated by unacceptable error.

One of the ways to bypass a problem of large difference in scale, would be a multiscale approach in which objects of considerably different scale size are modeled separately and those models are linked together with boundary conditions. That is how we approached the modeling of lubricating gap flow, including an energy equation in the model, since viscosity of fluid significantly influences the flow rate. We show how numerical simulation could enhance the design process of a novel type of high pressure axial piston pump.

## A Short Description of Axial Piston Pumps

A hydraulic pump is a mechanical device which transforms mechanical energy into hydraulic energy. It generates the flow of hydraulic fluid which is able to overcome the resistance pressure created by the load. There are several types of hydraulic pumps: gear, piston, rotary vane and screw pumps. The piston pumps could be divided into axial and radial pumps. Axial piston pumps could have a swash plate with variable swash angle or bent axis pumps.

### Piston Pumps

A piston pump transforms work of pistons that move in the reciprocating motion into pressure which is used to overcome external load through the driven fluid. Axial piston pumps have an odd number of pistons that move in cylinders encased in a pump body. Rotating cylinder block is driven by a shaft and through contact of a swash plate with piston slippers creates a reciprocating motion of pistons. Pistons suck oil from the inlet port and subsequently squeeze fluid creating pressure which drives oil into a high pressure port. Piston pumps can have variable or fixed displacement.

**Fig. 1:**
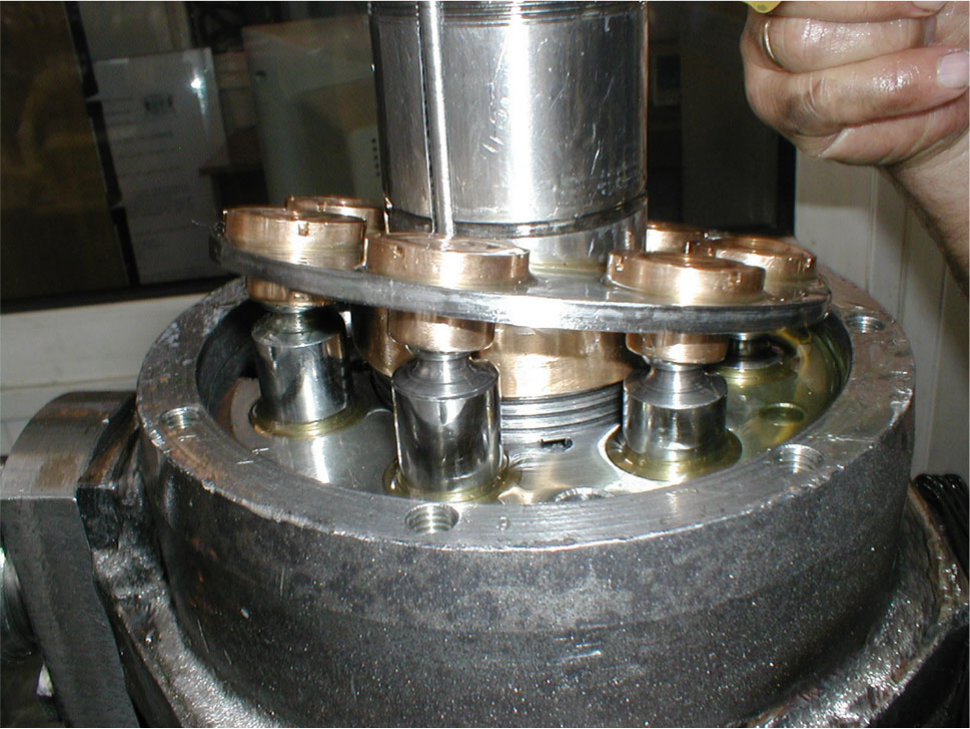
Swash plate on a novel piston pump with piston slippers—part of the PWK axial piston pump

In axial piston pumps pistons move in a reciprocating motion, parallel to a drive shaft. Fluid is controlled by the commutation unit. Efficiency of a fixed displacement pump is determined by a number and size of the pistons and the length of the stroke which in turn depends on the angle of the swash plate, cf. Fig. 1.

In variable displacement axial piston pumps, the angle of the swash plate can be adjusted through a movable yoke, which can be controlled manually or by servomotor or compensator.

In bent axis axial piston pumps—a shaft rotates a cylinder block with pistons, while the commutation unit and pressure ports are stationary. The shaft axis forms an angle with the cylinder block thus causing a reciprocating motion of pistons during the one revolution of the shaft. The pistons are sealed with rings like in a typical combustion engine.

This study deals with piston axial pumps with an angled swash plate.

### Lubricating Gaps in Axial Piston Pumps

**Fig. 2:**
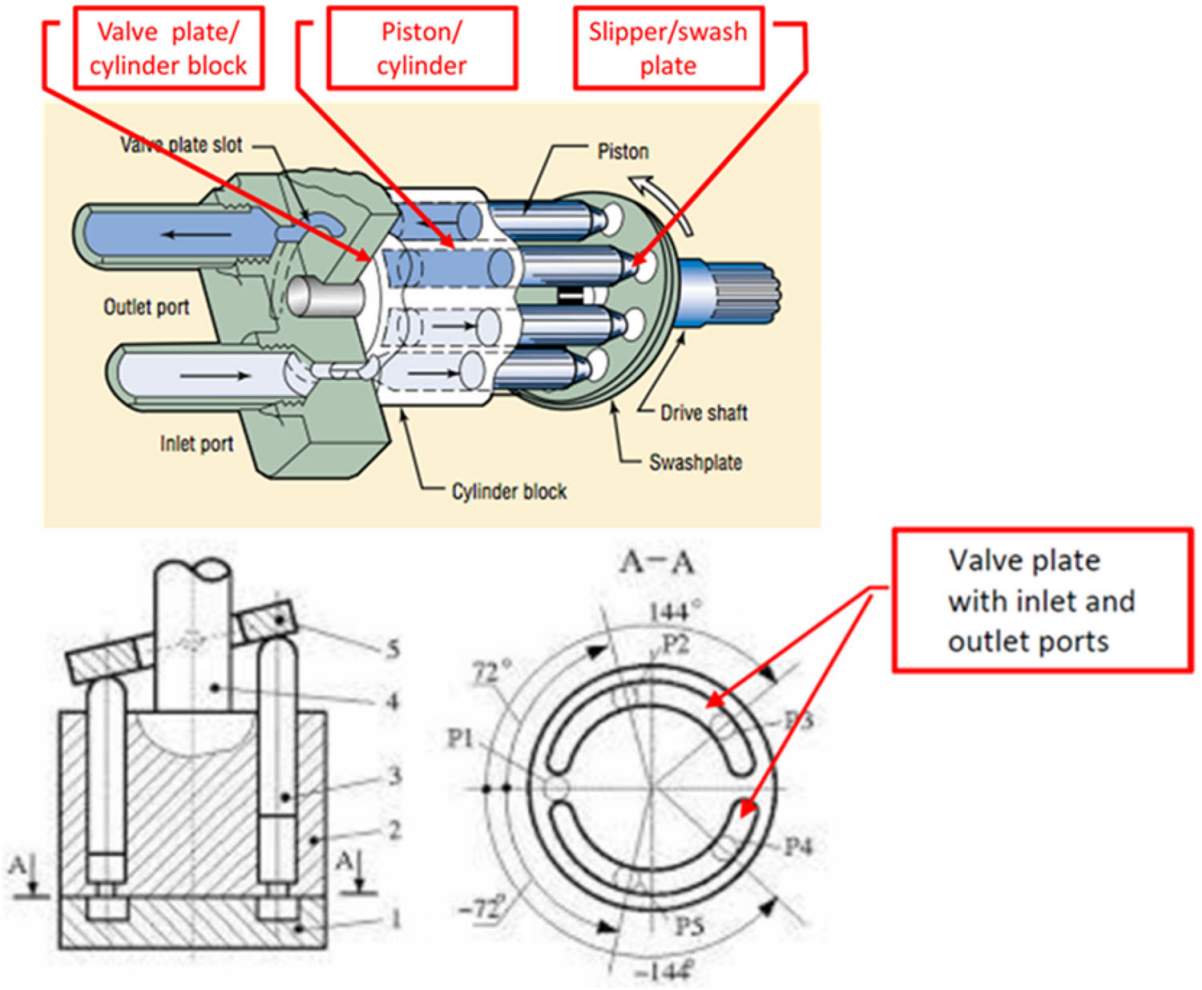
Traditional commutating port plate unit in axial piston pumps (*bottom*) and typical leakage locations (*top*)

Typical leakage locations in axial piston pumps are related to areas indicated in Fig. 2. Those gaps are essential for the operation of the pump since they provide lubrication between mating parts. The gaps could be described as having height of a few microns, while the fluid flow in the gaps is laminar and could be described by Navier–Stokes equations. The following assumptions are acknowledged: mass forces are neglected, flow is steady state, fluid velocity can change only in the direction of gap height and pressure is not a function of gap height.

### Published Research on Lubricating Gap Leakage in Axial Piston Pumps

Bergada et al. [1] studied hydrostatic leakage and lift characteristics of a slipper in a axial piston pump and motor. They developed a new set of equations for a slipper with any number of grooves. Bergada et al. [2] presented a complete analysis of piston pump flow losses, based on the model created from leakage equations in all gaps, which were tested on numerical models. The experimental data were tested against simulation results. A thorough research of studies, performed in the area of piston pump leakage was conducted. A problem of scale difference was tackled by Crompton et al. [3], where the Reynolds equation was implemented using the weak form. They used a model built in a finite element package COMSOL Multiphysics which defined a thin lubricant layer between two long solid walls. The Reynolds equation was defined as a boundary condition and no mesh was used in the model. Grabon et al. [4] used the finite difference method to calculate the distribution of hydrodynamic pressure in the radial bearing, applying the Reynolds theory of hydrodynamic lubrication. Hao and Qi [6] analyzed the effects of leakages on the loss off low rate and pulsing of the axial piston pump. Ivantysynova [7] summarizes the main contributions to the discovery of physical phenomena defining the fluid film properties and the operational conditions of the piston cylinder interface in hydrostatic piston machines. She attaches a sizeable list of references. Ivantysynova and Huang [8] presented a method for complex gap flow simulation of connected and selfadjusting gaps of axial piston machines, where the model for the piston/cylinder pair was extended to consider elastohydrodynamic effects. Kumar [9] in his PhD thesis conducted CFD analysis of the piston axial pump. Marsalek et al. [11] presented the modelling of a powertrain slide bearing incorporating mixed lubrication conditions. Osiecki and Patrosz [14] described the capacity varying mechanism of the novel PWK piston axial pump and analyzed sources of leakages in that pump. Qiong et al. [16] created an engine virtual prototype using finite element method and multi-body dynamics method, used elastohydrodynamics theory and solved Reynolds equations and established that oil film dynamic lubricating friction effected dynamic simulation calculations. Sinha et al. [17] studied the effect of the viscosity pressure exponent, on various characteristics of the bearing has been studied. Sorsimo et al. [18] solved the inequality constrained Reynolds equation using the finite element method. The pressure profiles were similar to those obtained from the experiments and the contact area was found accurately, but it was an overestimate compared to the cavitational region found in the experimental tests. Stevanovic et. al [19] derived general slip-corrected Reynolds lubrication equation and showed that it possesses an exact analytical solution. Wieczorek and Ivantysynova [20] developed a method to calculate non-isothermal gap flow in the connected gaps of swash plate type axial piston machines by combining Matlab and C language routines that analyzed the motion equation of all moveable parts of the rotating group and performed gap simulation of all concerned gaps. Haynes in his dissertation [21] performed piston pump leakage modeling and measurements. Zloto [23] calculated the pressure distribution of the variable height gap between a valve plate and cylinder block using a finite analysis program he developed.

### Description of PWK Pumps

Axial pumps with cam-driven commutation units—so-called PWK pumps—emerged as a result of a research project conducted in the Department of Hydraulics and Pneumatics at the Gdansk University of Technology [6].

**Fig. 3:**
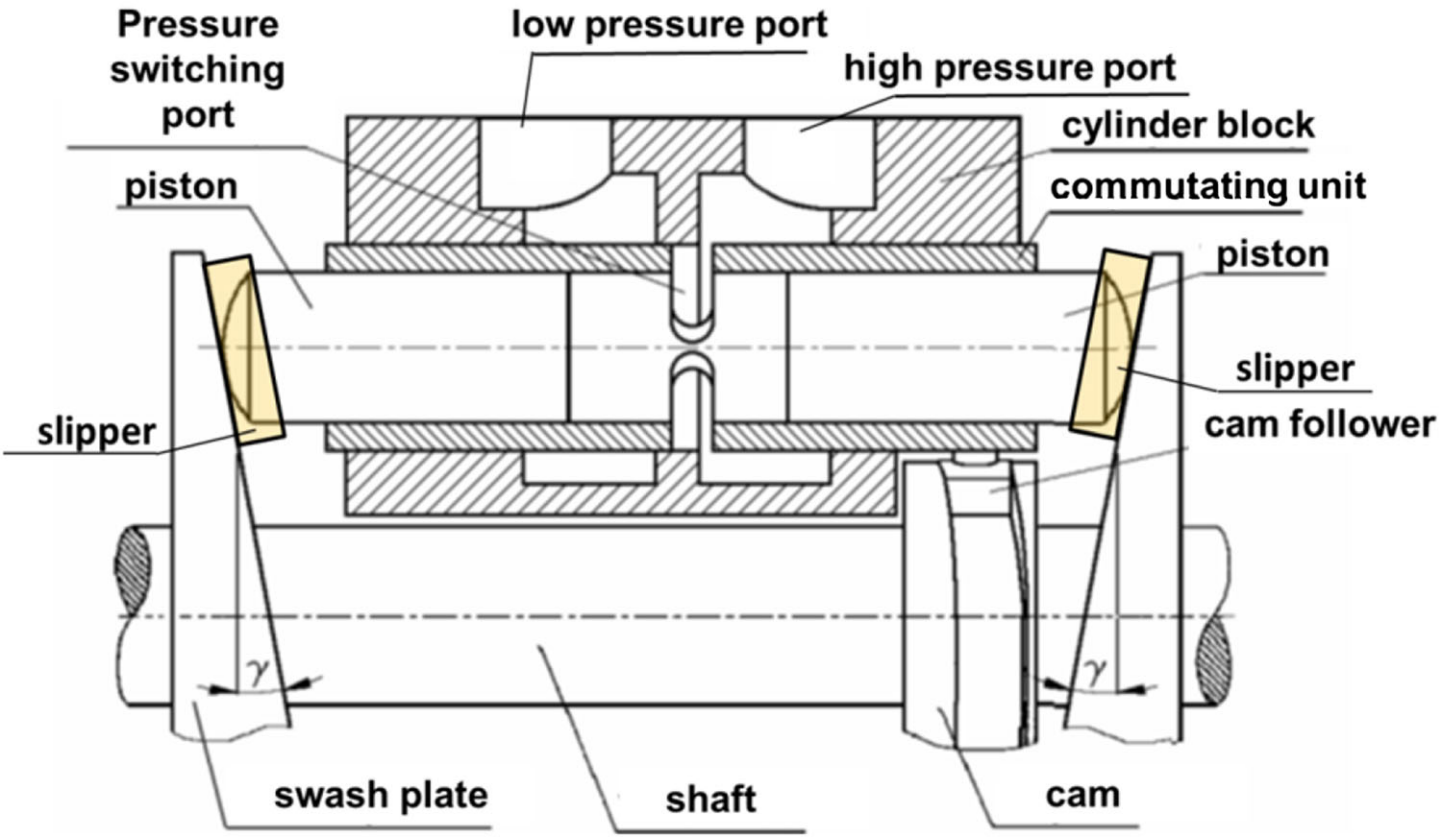
Simplified cross section of the control mechanism of the PWK pump

As for all axial hydraulic piston pumps several cylindrical working chambers are positioned around the rotating shaft of the pump. The rotation of the shaft and the attached swash plate leads to the reciprocal movement of the pistons which decreases and increases the fluid volume of the chambers alternately. A window (or a pressure-switching port)—which is part of the control sleeve or the commutating bushing—connects alternately the chamber between the piston faces with low and high pressure channels, cf. Fig. 3.

**Fig. 4:**
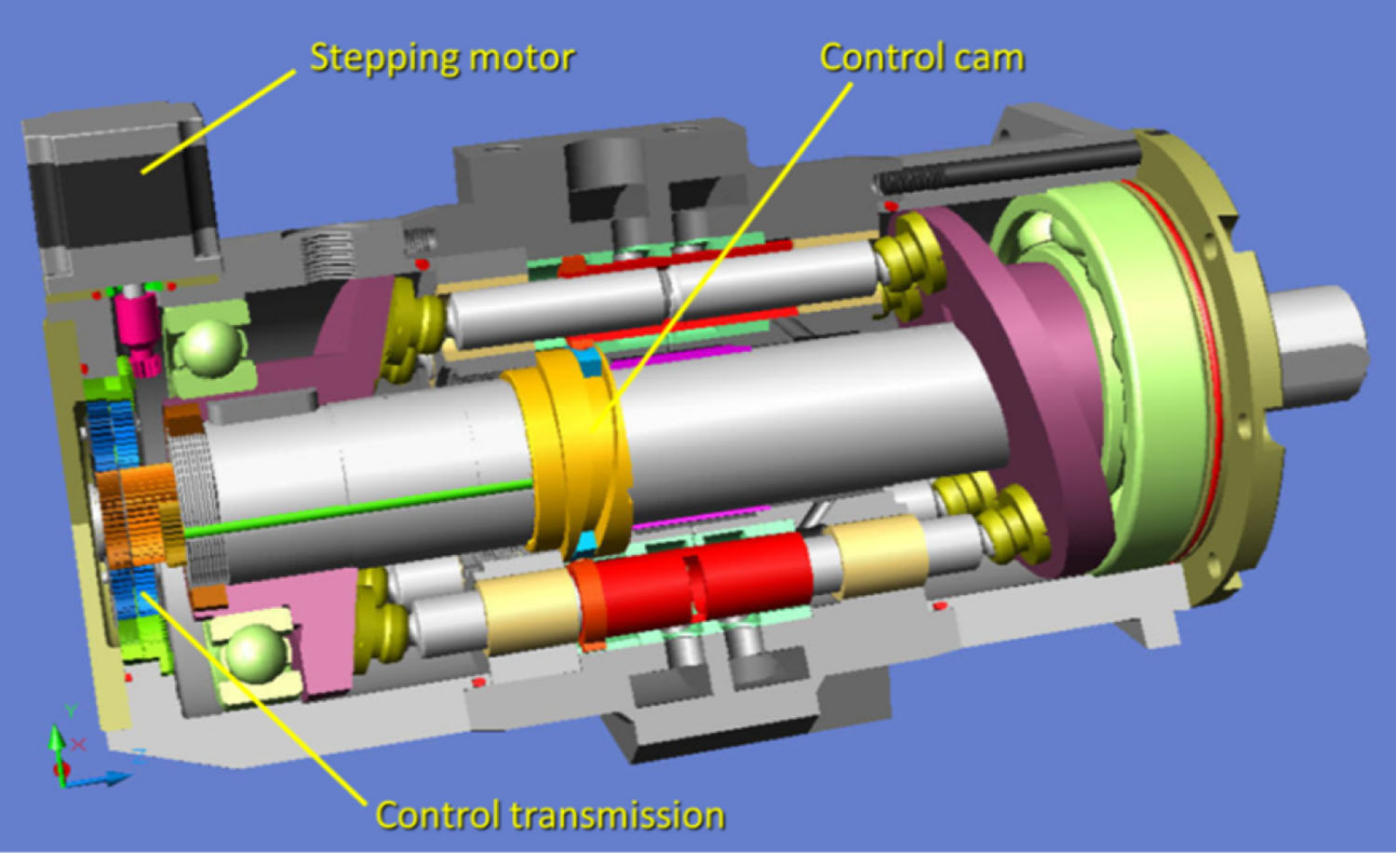
Variable displacement PWK pump

The axial piston pumps with constant displacement show a very good performance with a working pressure of up to 55 MPa, an overall efficiency of 94 % and good power density (Fig. 4).

Although the pistons of the PWK pump always travel the same distance inside the displacement chamber, the pump’s overall displacement may be controlled by changing the angular position of the control cam in relation to the pump’s shaft. As both parts are rotating fast a special planetary gearbox, that ensures precise control over the displacement in the operating range, was developed. Thanks to hydrostatic discharge, such a mechanism requires low input energy and can be driven by a stepping motor. This is the major advantage of the newly developed pumps over all other variable displacement pumps, controlled with a complicated hydraulic servomechanisms. It reduces the pump’s cost and dimension significantly, thereby simplifying the operation and boosting the reliability.

PWK pump is a new development. Different features of the pump need to be investigated using prototypes and numerical simulation. The experimental results need to be compared with the results of the numerical simulation. With a validated simulation model different configurations of the new pump can be tested without expensive experimental testing. An example of such simulation is shown in Sect. 2.6, where a compensation chamber is added to the existing pump design and different parameters of the pressure relief channels are tested in order to find the optimal configuration of the compensation chamber (Table 1).

**Table 1:** Two test configurations compared

Test configuration	Number of passages	Diameter (mm)	Membrane thickness (mm)
Configuration 1	1	1	1
Configuration 5	2	1.4	1

### 3D CFD Model of an Axial Piston PWK Pump

**Fig. 5:**
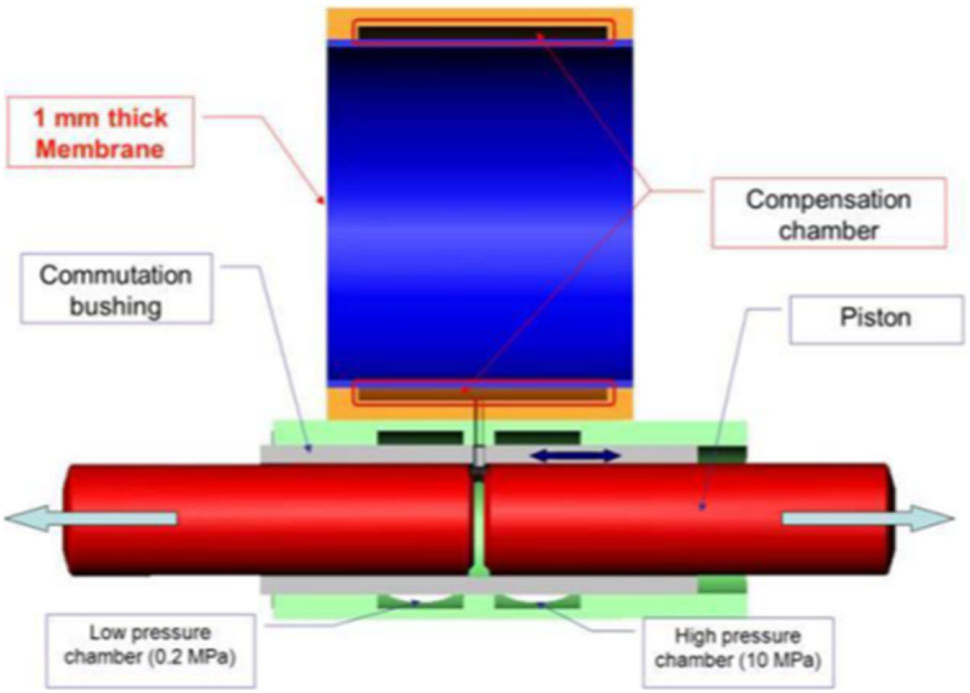
Detailed 3D CAD model of the pump that served as reference to generate a CFD model

Computer modeling became a standard tool used throughout a wide spectrum of engineering applications. Its unquestionable advantage is the ability to predict operational states of the design under consideration, even before building the working prototype. One of the fields specially sought after is computational fluid dynamics (CFD) (Fig. 5).

The geometric solid model of the pump was used to create the “negative image” of its unfilled pores and spaces—the voids were given volume and the pump elements were removed. Therefore the true volume of fluid was extracted from the original model. The simulation model approximates the physical model of a working pump. In reality the cylindrically shaped pump body houses seven pairs of pistons, which are equally spaced around the perimeter of the body.

**Fig. 6:**
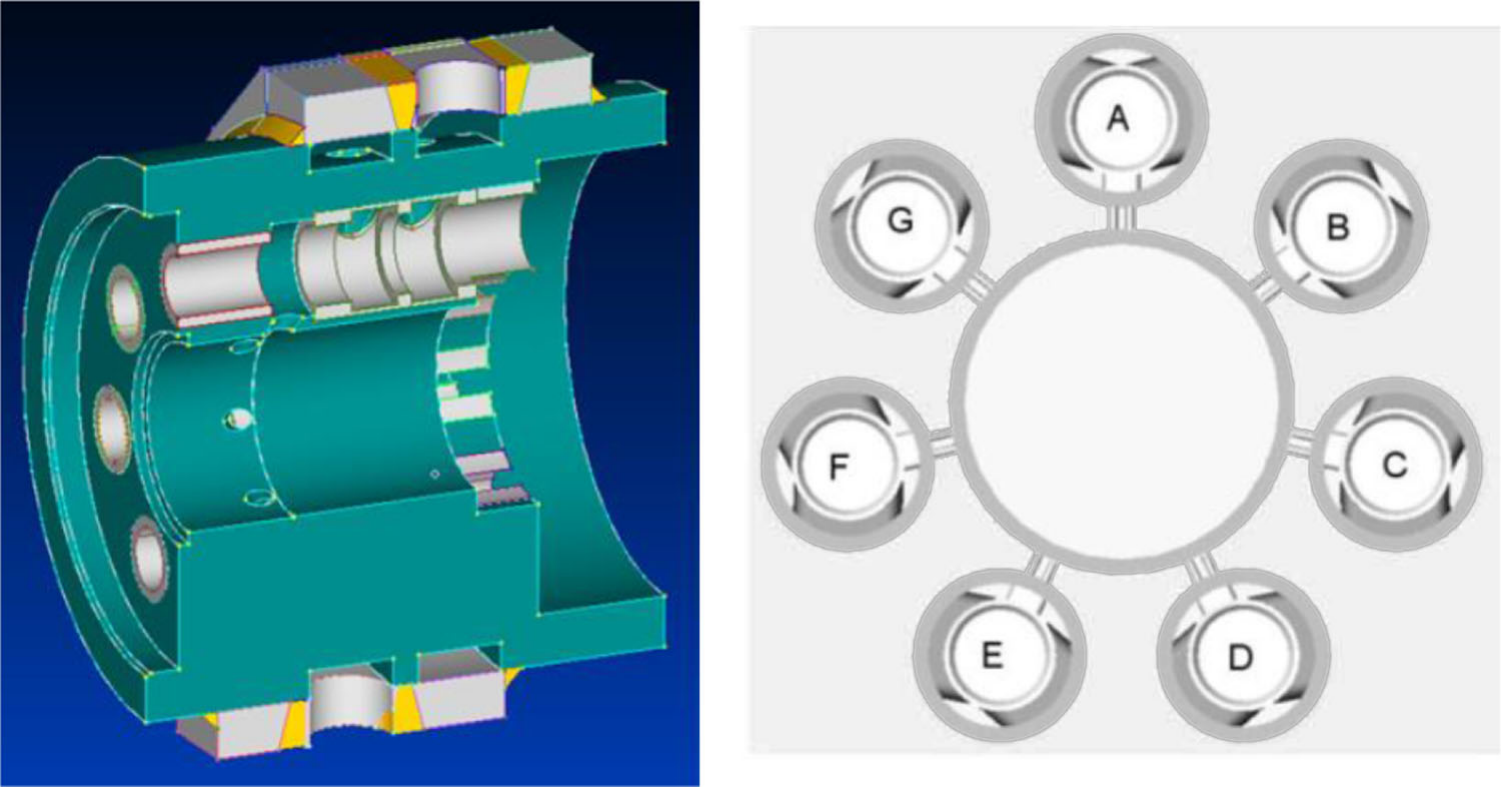
CAD model of a pump body (*left*) and a sketch of seven displacement chambers (*right*)

Since the size of the model can heavily influence the calculation time there were several steps taken in order to make the fluid model more compact. A symmetry of the system was taken advantage of, which allowed to limit the number of working chambers from 7 to 2. Since the pump has seven working chambers evenly spaced around the perimeter of the pump body, the approximation was used in a CAD model—by shifting a chamber called D, normally placed at 5 o’clock on the perimeter of the pump (Fig. 6) to a 6 o’clock position, opposite to chamber A. The whole pumping cycle takes place during one shaft revolution with all seven pairs of pistons and commutating bushings synchronized accordingly, therefore the original phase shift was used in a symmetric model.

**Fig. 7:**
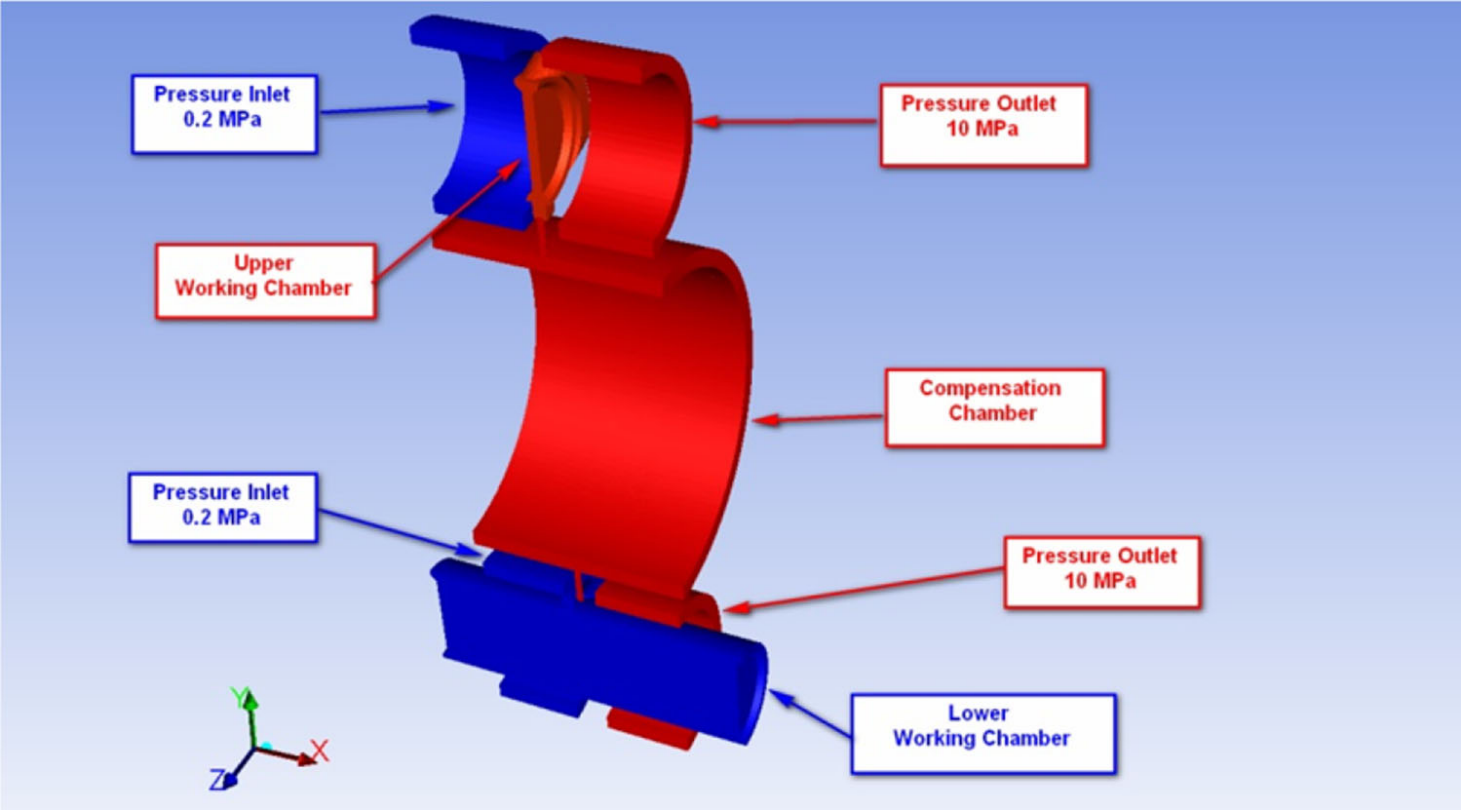
Simplified symmetrical CFD model of a fluid showing upper and lower chambers only (2 out of 7)

The operation of the pump was simulated with the Fluent CFD code. Several models of the pump were constructed: a full (global) model for verification of data, simplified models consisting of 2/7 of the pump as well as local models of gap flow. Each one was used for a different purpose at a different stage of analysis. A volumetric mesh was generated—it was built from hexagonal and tetrahedral elements. The Navier–Stokes equations were used to define the motion of hydraulic fluid in the pump. Since the model was to capture the dynamic behavior of working fluid, the moving dynamic mesh (MDM) concept was used in Fluent. It allows a model to deform its mesh by extending it or retracting the boundaries, according to an assigned equation of motion. The presented example is based on a particular design case: the variable capacity pump was set to its 100 % capacity and the shaft was revolving at 1000 rpm. Fast moving parts (pistons with the velocity of about at 1.3 m/s and a commutating bushing moving at 0.5 m/s) and the small size of elements defined the length of the integration step, which was set at the 1e^−4^–1e^−5^ s, depending on the size of the model. That was essential to capture the propagation of pressure waves in the working fluid. The computational results were compared to test readings and the length of the integration step was confirmed as correct and adequate, although it is demanding on computer power. The fluid model shown in Fig. 7 consists of 140,286 volume cells and 166,315 nodes.

### Modeling Pump Leakage

Oil leakage in the PWK axial piston pump could be classified into two categories: pressure-relief—introduced purposefully by designers and lubrication gap leakage, typical for axial piston pumps. The pressure-relief leakage was to compensate pressure peaks created by the pressure of fluid, when the working chamber would be cut off from any pressure-relief port (inlet or outlet) and pistons would complete compressing of fluid. Therefore to eliminate the negative effects that excessive pressure might cause (high noise, risk of damage to internal parts) a sleeve surrounding the drive shaft was squeezed into the cylindrical block, thus creating a cavity that could absorb extra fluid or inversely, could serve as a supply of fluid to chambers around the perimeter of the cylinder block, when needed [10, 15]. Each out of seven displacement chambers was connected with the compensating chamber by means of a narrow passage. A study on the passage configuration was conducted in order to optimize pressure in all chambers. Simulation was performed to find an optimal pressure-relief hole configuration, taking into account the hole diameter and the number of holes. A fluid–structure interaction (FSI) model was created. Parameters resulting from analysis of fluid conducted in a CFD package (Fluent) were passed on to a finite element package—Ansys, where the reaction of the membrane was calculated and passed back to Fluent. There were two major variations in tested models: the number and size of relief passages and the thickness of the membrane, which effected the volume capacity of the compensation chamber.

**Fig. 8:**
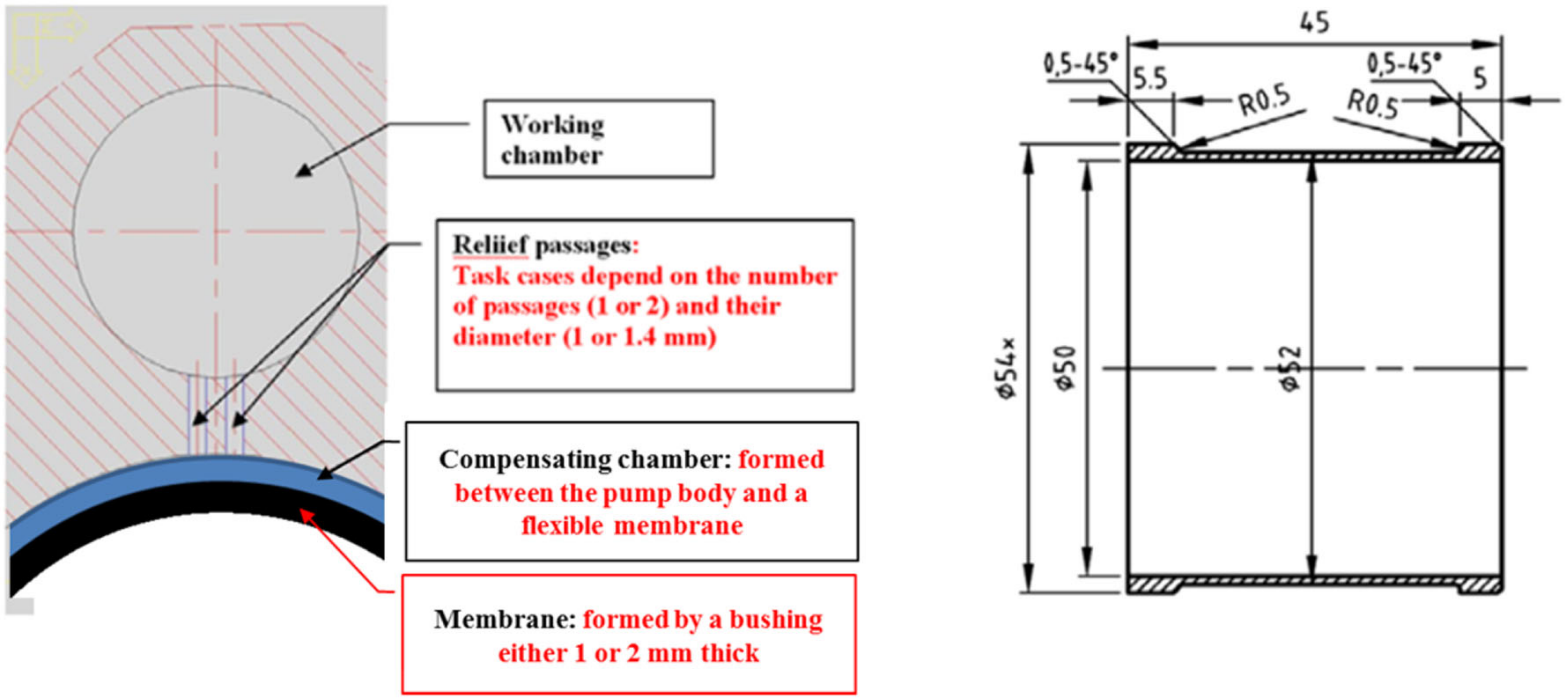
Task definition for relief hole optimization: relief hole configuration (*left*) and the proposed membrane (*right*)

The diameter of pressure-relief holes was varying from 1 to 1.4 mm and the number of holes for each size was changing from 1 to 2 (Fig. 8).

**Fig. 9:**
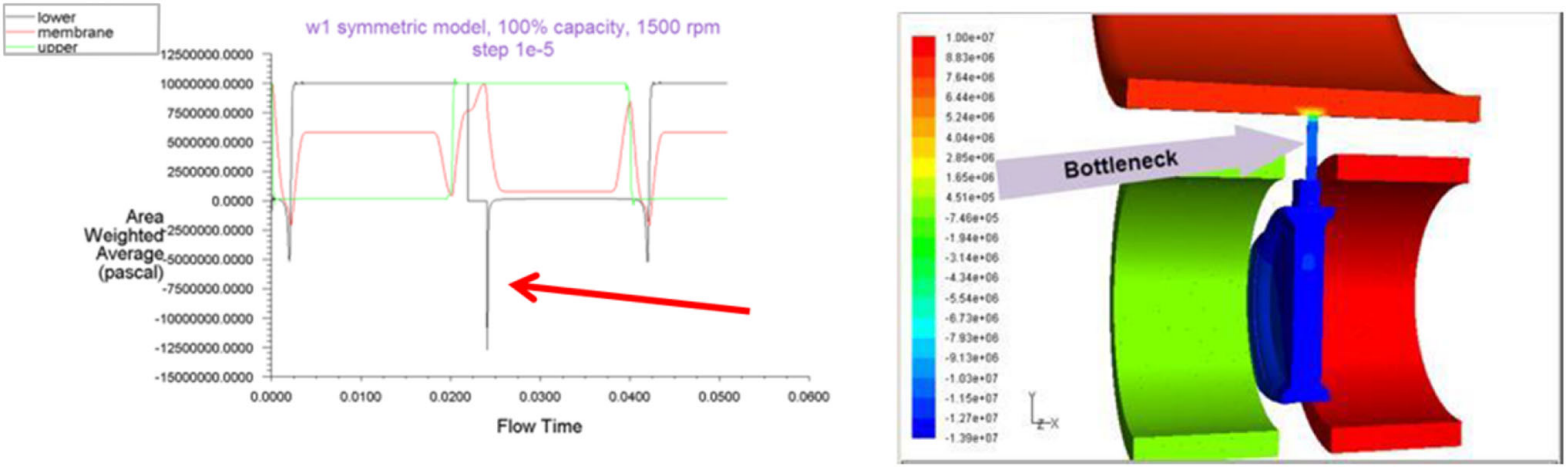
Pressure drop in a model with one relief hole of 1 mm diameter

Simulation conducted on a model equipped with one relief hole indicated a visible negative pressure peak which stipulated the restriction of the flow by insufficient area of the passage. That can be clearly seen in Fig. 9.

The next task concentrated on the analysis of a model equipped with an additional relief hole.

**Fig. 10:**
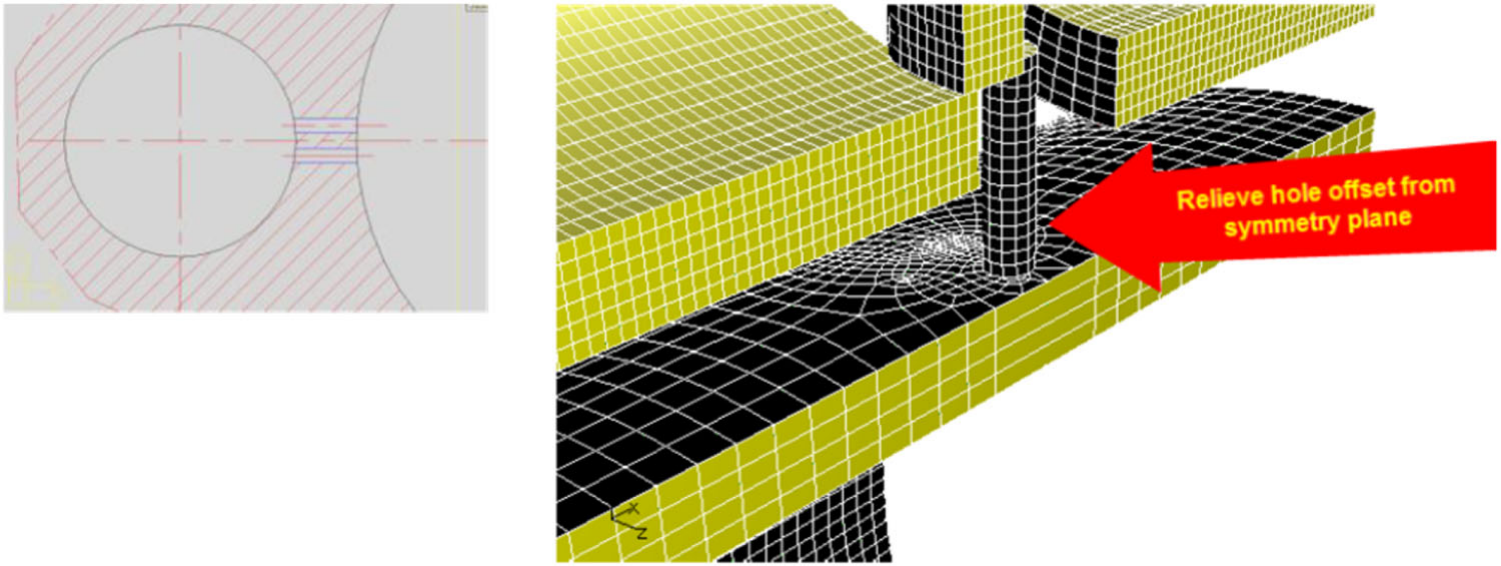
Model with two relief holes connecting displacement and compensating chambers

Figure 10 displays a symmetric half of the complete model, thus displaying only one pressure-relief passage.

**Fig. 11:**
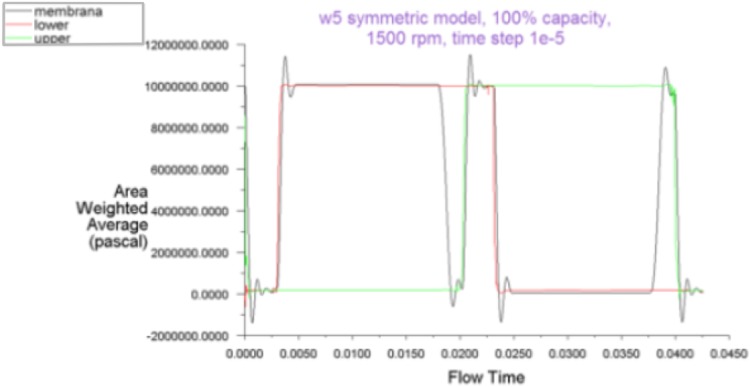
Pressure plot for the model with two holes of 1.4 mm

The addition of an extra hole and the increase in the hole diameter eliminated pressure peaks (Fig. 11).

**Fig. 12:**
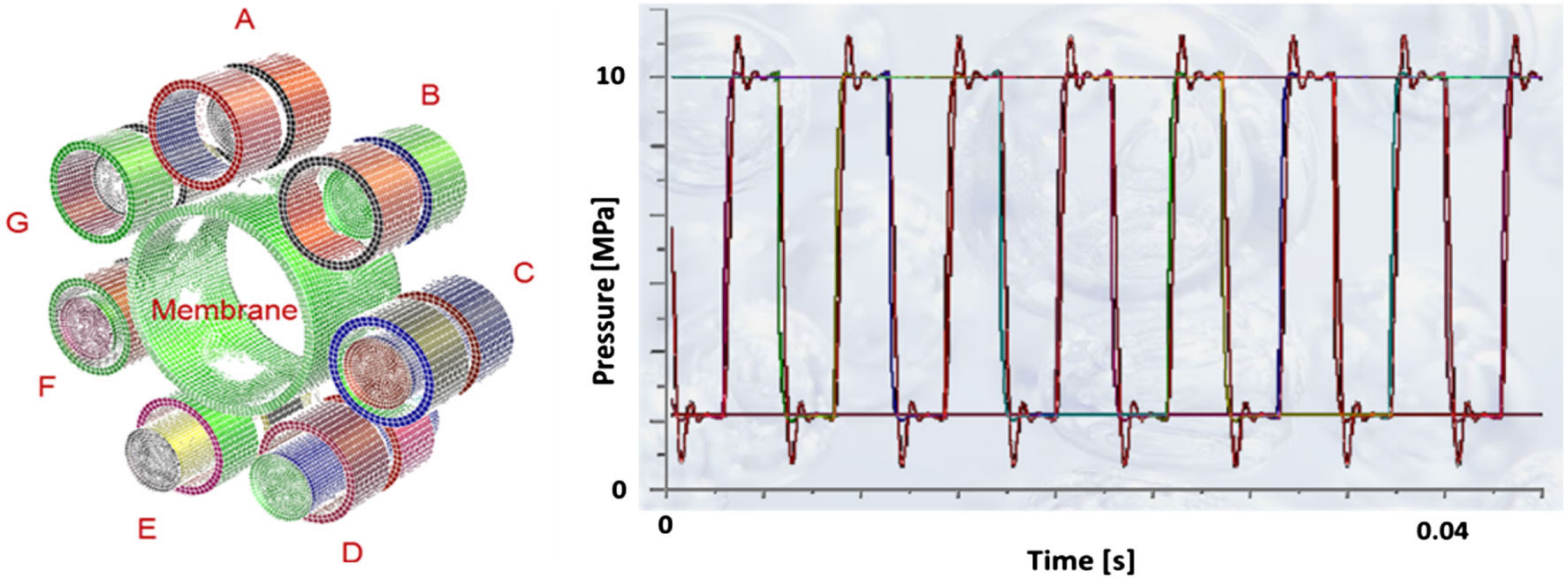
A CFD model of a full pup (*left*) and pressure plot of a full 7-chamber model with two relief holes (100 % displacement, 1500 rpm) (*right*)

Finally the full model of the pump was created in order to validate the results received from the analysis with the use of a symmetric model. Figure 12 shows the pressure plot in all seven chambers of PWK pump equipped with two pressure relief holes in each displacement chamber. Its good agreement with results received from a simplified symmetric model of the pump shows that smaller models offered acceptable approximations, which simplifies the analysis process.

### Leakage in the Lubricating Gaps of the PWK Axial Piston Pump

The analysis that follows refers to a paper by Osiecki and Patrosz [14] and could be considered as an extension of their study to assess leakage in lubricating gaps of the PWK axial piston pump.

**Fig. 13:**
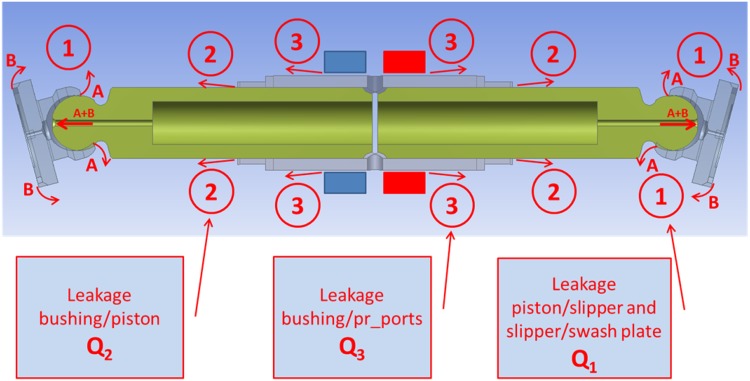
Location of gap leakage in the PWK axial piston pump

In contrast to typical axial piston pumps equipped with the traditional port-plate commutation units, cf. Fig. 2, there is one difference in the location of lubricating gap leakage as far as PWK pumps are concerned, namely the occurrence of the gap between the commutation sleeve and pressure ports, shown in Fig. 13 as Q_3_.

There are four lubricating gap leakages found in PWK axial piston pump:

- Between a slipper and a swash plate.
- Between a slipper and a piston.
- Between a piston and a commutating bushing.
- Between a commutating bushing and pressure ports.

Because flows from two first leakage cases add up to one leak through a helical gland the proposed models were classified as follows:

- Q_1_—Leakage through the helical gland equal to the sum of leakages between a slipper and a piston and slipper and the swash plate.
- Q_2_—Leakage in the gap between a piston and commutation sleeve.
- Q_3_—Leakage between a commutation sleeve and high and low pressure ports.

Model of lubricating gaps was created with the assumption that boundary conditions imposed on the local model will be based on data calculated in the global model. The creation of the local model was motivated by difference of scale between the dimensions of the displacement chamber and the size of the lubrication gaps. The local model consisted of the mesh of volume elements which define the laminar flow of incompressible fluid. The CFD model of fluid in PWK pumps, which was used in earlier analyses did not take into account leakages. To optimize calculations the model consisted of only two displacement chambers connected with the compensation chamber. The results of calculations showed good compatibility of the limited model with the full model. Three leakage models were selected for analysis, as shown above. The biggest of them all was connected to the leakage under the slipper, which was equivalent to the leakage in the helical gland. The simplified model of the pump was using symmetry of the structure to decrease the size of the model. Symmetry of the model was disturbed by the helical shape of the groove placed on the perimeter of the gland. Therefore the model had to include the rest of the pump so far neglected due to symmetry. Its size grew to 1.3 M volume cells. CFD software package Fluent, with parallel processing module was used taking advantage of 24 processors. The local model which comprised the leakage model in a helical gland was one order of magnitude smaller.

The global model of the pomp can be characterized as follows:

- It is limited to two displacement chambers connected with the compensation chamber which serves as a pressure equalizer.
- Fluid mesh is dynamically changing its volume in time.
- Density of fluid is dependent on pressure and controlled by the user defined function.
- Model does not take into account the elastic deformation of the structure.
- Energy equation is considered.
- Viscosity dependence on temperature is taken into account.

Local models of the pump were created separately for each of described cases. They share common features:

- Flow is laminar.
- Local boundary conditions are intercepted from the global model.
- Model provides results like: volume flow rates, pressure, velocity and temperature fields.

The following local models were created: for case no. 1 (leakage between a piston and a slipper) an equivalent 3D model of a flow in a helical gland groove was created. Additionally because of the similar dimensional scale (a groove in a gland has a width of 0.5 mm, which differs dramatically from the size of other gaps, whose average height is 12 µm), the global model was supplemented by the helical gland leakage in order to compare results obtained from the local model. They agreed fully.

For case no. 2, which simulated leakage in a gap between a commutation bushing and a piston—two models were created: an axisymmetric model which corresponded to the concentric location of a piston in a cylinder and a 3D model of a developed geometry of the gap, that defined a skewed piston in a cylinder.

The third case defined a leakage between a commutation bushing and pressure ports of the pump. It was simulated by an axisymmetric model of the gap, assuming a concentric position of a bushing in a cylinder formed by the structure of the pump containing low and high pressure ports. Models included the following parameters: low pressure set at 0.2 MPa, high pressure equal to 10 MPa, rotational speed of the drive shaft equal to 1000 rpm, pump capacity was set at 100 %.

The properties of hydraulic oil used in PWK pumps, Azola ZS 46 are shown in Table 2.

**Table 2:**
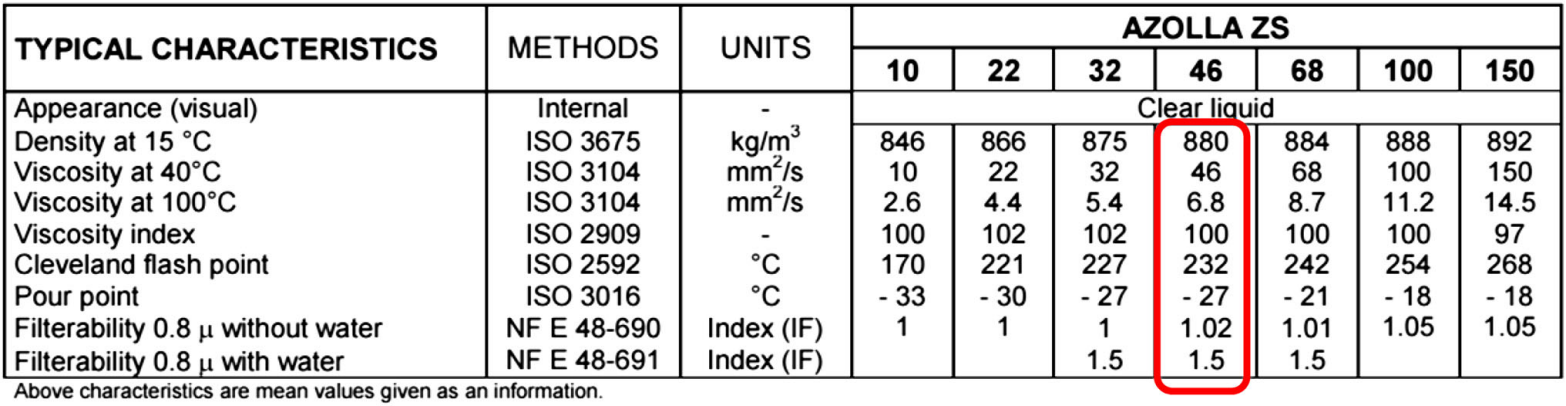
Properties of Azolla ZS 46 hydraulic oil used in PWK pumps

#### Equations of Motion

If ***u*** denotes the fluid velocity, ***f*** the total of body forces, ***T*** the stress tensor, ***ρ*** the constant fluid density and $\frac{D}{Dt} = \left( {\frac{\partial }{\partial t}} \right) + \varvec{u} \cdot \nabla$ the material derivative, then the equations governing the flow of an incompressible fluid are

1 $$\nabla \cdot \varvec{u} = 0$$

2 $$\varvec{\rho}\frac{{D\varvec{u}}}{Dt} = \rho \varvec{f} + \nabla \cdot \varvec{T}$$

The constitutive equation, relating the stress tensor to the rate of strain tensor, for an incompressible Newtonian fluid is $\varvec{T} = - p\varvec{I} + \mu \nabla \varvec{u}$. Thus, in the absence of body forces, the basic set of equations governing the flow of an incompressible fluid becomes

3 $$\nabla \cdot \varvec{u} = 0$$

4 $$\frac{{\partial \varvec{u}}}{\partial t} + \left( {\varvec{u} \cdot \nabla } \right)\varvec{u} = - \frac{1}{\rho }\nabla p + \nu \nabla^{2} \varvec{u}$$

where *p* denotes the fluid pressure, *μ* dynamic viscosity and $\nu = \frac{\mu }{\rho }$ the kinematic viscosity.

#### Piston End-Related Leakages

**Fig. 14:**
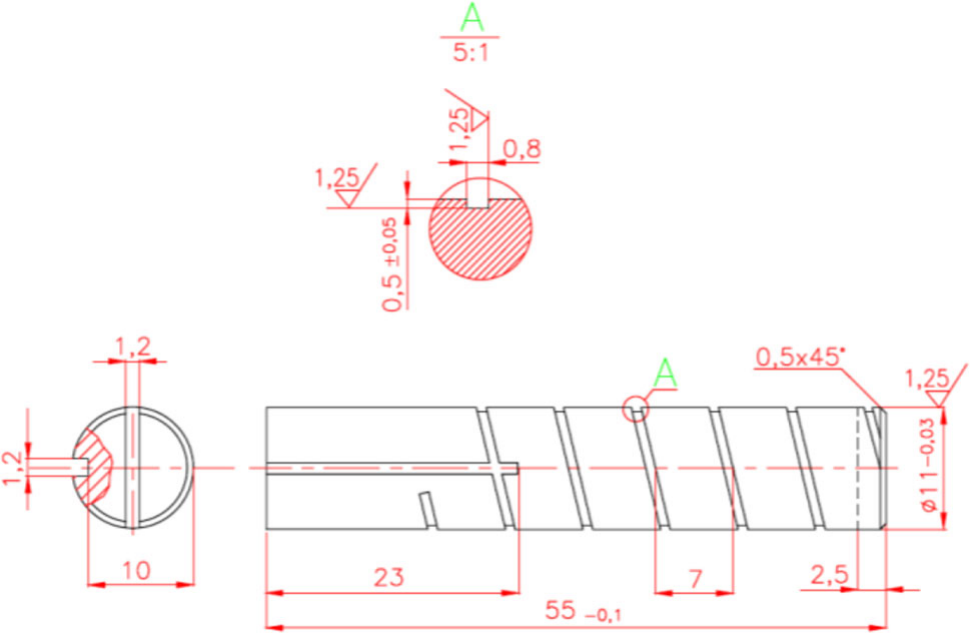
Drawing of a helical gland inserted in the piston

One of the critical elements of the axial piston pump is the slipper which transmits forces between each piston and a swash plate. To assure its continuous operation, constant lubrication between the slipper and the swash plate has to be provided. It is realized by means of oil flowing in a channel within each piston. Pistons are hollow and filled with mating helical glands. Glands are the components of the hydraulic system which are responsible for the change of flow rate or decrease of fluid pressure. Details of the helical gland used in PWK pumps are shown in Fig. 14.

**Fig. 15:**
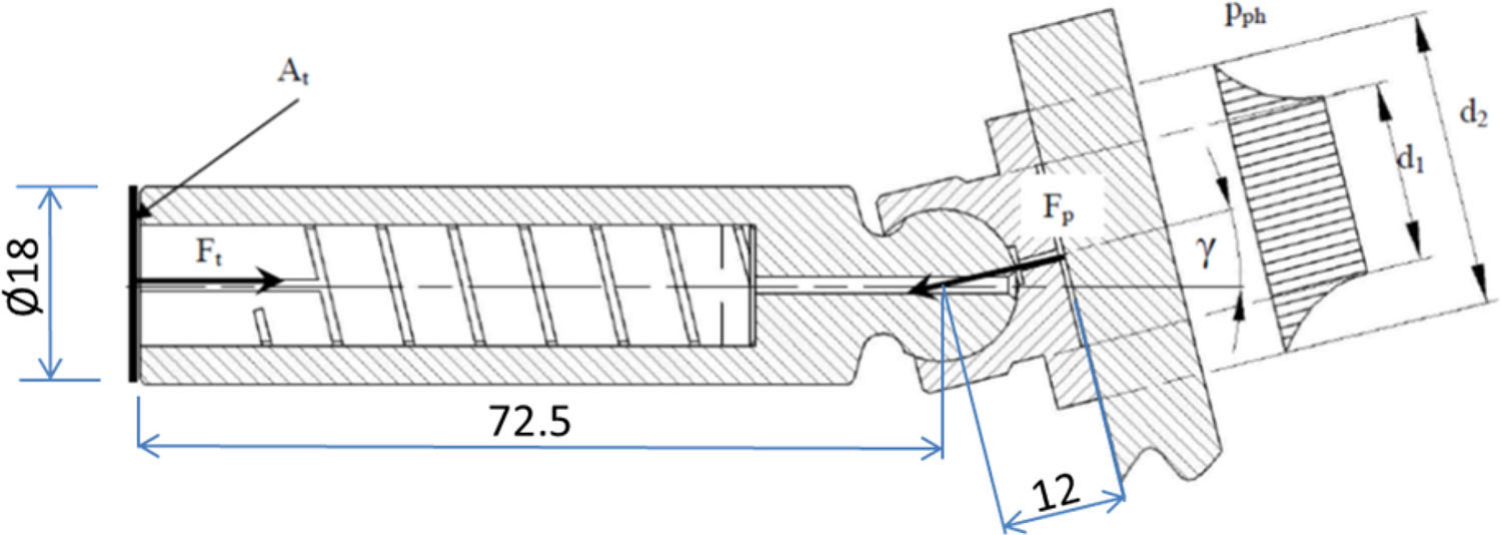
Pressure distribution under the slipper of piston axial pump PWKZ

Oil leaves the displacement chamber and upon following the helical path around the perimeter of the gland enters the area between the slipper and the piston and between the slipper and the swash plate. Since both of those leakages are supplied by the flow of fluid from the helical gland and owing to the fact that the latter was already modeled as part of the global fluid model, it was subsequently extracted from the global model and analyzed (Fig. 15).

**Fig. 16:**
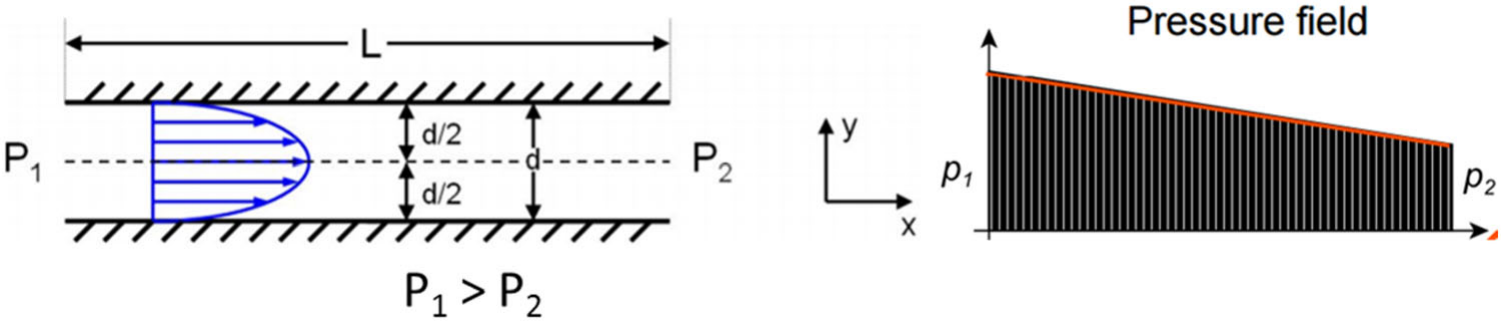
Poiseiulle flow between parallel plates

The flow through the helical gland could be considered as Poiseuille flow, with both plates stationary (Fig. 16).

Velocity of flow can be calculated with the following formula:

5 $$v = \frac{1}{2 \times \mu }\left( {\frac{\partial p}{\partial x}} \right)\left( {\frac{{{\text{d}}^{2} }}{4} - x_{2}^{2} } \right)$$

where *μ*—dynamic viscosity (Ns/m^2^), *d*—gap height (m).

The average velocity can be derived from:

6 $$v_{ave} = \frac{{\Delta Pd^{2} }}{{12 \,\upmu{\text{L}}}}$$

Assuming the gap of height *h* = 0.0005 m, Δ*p* = 2,799,248 Pa, *L* = 0.166 m and *μ* = 0.026 P s, gives *ν*_*max*_ = 19.1 m/s, *ν*_*average*_ = 13.5 m/s.

**Fig. 17:**
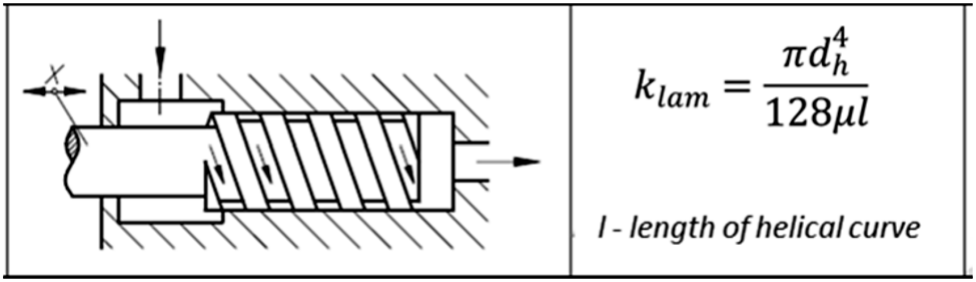
Flow rate coefficient estimation applied to the helical gland

For laminar flow, the volume flow rate through a helical gland could be calculated according to a formula:

7 $$Q = k_{lam}\Delta p$$

where the coefficient *k*_*lam*_ can be estimated from Fig. 20 below, bearing in mind that $\frac{L}{{d_{h} }} > 20$ and *Re* < 2300 (Fig. 17).

In its general form the Poiseuille equation could be presented as follows:

8 $$\Delta {\text{p}} = 8\,\upmu{\text{Q}}\frac{{\Delta {\text{x}}}}{{\uppi{\text{r}}^{4} }}$$

**Fig. 18:**
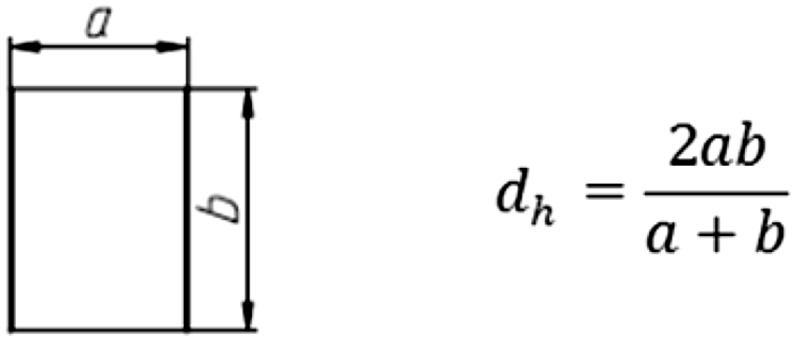
Hydraulic diameter formula

Pressure difference Δ*p* between the displacement chamber and a hydrostatic support (slipper) can be calculated from formula (9) as

9 $$\Delta p = p - \frac{{8 \cdot p \cdot A_{t} }}{{\frac{{d_{2}^{2} - d_{1}^{2} }}{{\ln \left( {\frac{{d_{2} }}{{d_{1} }}} \right)}} \cdot \pi \cdot cos\gamma }}$$

where *p*—the pressure in the displacement chamber; *A*_*t*_—area of the piston; *d*_1_ and *d*_2_—characteristic diameters of the slipper (cf. Fig. 18); *γ*—swash plate inclination angle.

Assuming that *d*_1_ = 16.5 mm, *d*_2_ = 27 mm, *A*_*t*_ = 2545 mm^2^, *γ* = 14° and *p* = 10 MPa, then pressure under the slipper is 7,200,751 Pa and Δ*p* = 2,799,248 Pa. Calculating *k*_*lam*_ gives: 8.14e^−13^ m^4^s/kg, with r = 0.0005 m, l = 0.166 m, *d*_*h*_ = 0.0006 m and *μ* = 0.026 kg/ms.

Volumetric flow rate *Q* = 8.14e^−13^ × 2,799,248 Pa = 2.28e^−6^ m^3^/s or 0.134 l/min.

An alternative way to calculate the volumetric flow rate is offered by [12]. In cases where the width of a gap b is comparable with its height h (b/h ≤ 10), forces acting on side surfaces of the lubricating film have a significant influence on the flow resistance. In such a case a correction coefficient *k* has to be introduced in a formula defining the volumetric flow rate:

10 $${\text{Q}} = \frac{{\left( {\Delta {\text{p}}\,{\text{b}}\,{\text{h}}^{3} } \right)}}{12\mu l } k$$

where *Q*—volumetric flow rate (m^3^/s); *b*—gap width (m); *h*—gap height (m); *μ*—dynamic viscosity (kg/ms).

The correction coefficient k could be used according to Table 3.

**Table 3:** Correction coefficient to be used in calculating flow rate in rectangular gaps

b/h	∞	10	5	3	2	1
k	1	0.94	0.88	0.79	0.69	0.42

Now, using the previously defined variables and adjusting the correction coefficient to match the width to depth ration = 1.6 one gets the following result:

11 $$\begin{aligned} {\text{Q}} = \frac{{\left( {\Delta {\text{p}}\,{\text{b}}\,{\text{h}}^{3} } \right)}}{12\mu l } k & = \frac{{2799248\,{\text{Pa}} \cdot 0.0008\,{\text{m}} \cdot 0.0005^{3} \,{\text{m}}^{3} }}{{12 \times 0.026\,{\text{kg/ms}} \cdot 0.166\,{\text{m}}}} \cdot 0.552 \\ & = 2.98{\text{e}}^{ - 6} \,{\text{m}}^{3} / {\text{s}} = 0.179\,{\text{l/min}} \\ \end{aligned}$$

Using the output from (11) the flow velocity can be calculated as

12 $${\text{v}} = \frac{\text{Q}}{\text{A}}$$

Since *Q* = 2.98e^−6^ m^3^/s and *A* = 4.26e^−7^ m^2^, thus

13 $${\text{v}} = 7\,{\text{m/s}}$$

**Fig. 19:**
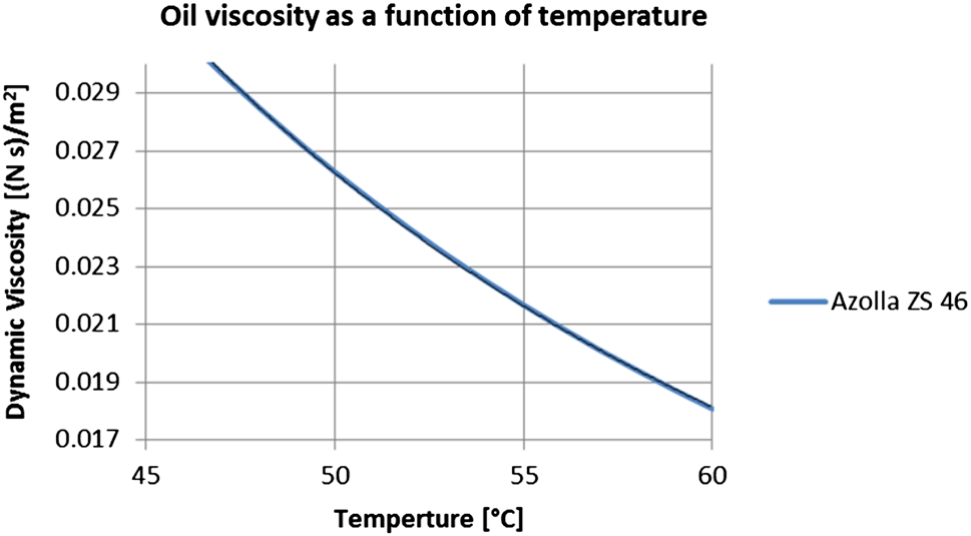
Dynamic viscosity of hydraulic oil as a function of temperature

Since viscosity is the function of temperature and that changes with the change of pressure in the lubricating gap, viscosity was coded into a user defined function according to the plot shown in Fig. 19.

#### Model Description

Assumptions:

- 3D model of a leakage between a slipper and a piston.
- Heat input (oil temperature at 49 °C and temperature near the slipper at 60 °C).
- Viscosity controlled by user defined function (dependent on temperature).
- Density controlled by user defined function (dependent on pressure).
- Input pressure controlled by user defined function.
- Output pressure at controlled by user defined function.

**Fig. 20:**
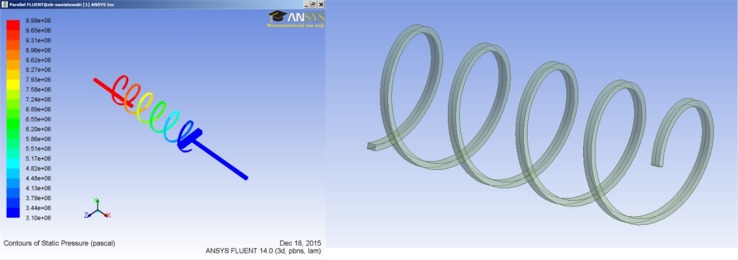
Model of flow though the piston (*left*), and its extracted helical path (*right*)

In order to have a clear reference to analytical calculations obtained from a model of an internal leakage through a piston, a submodel of that leakage flow was extracted—limited to a helical path only (Fig. 20).

**Fig. 21:**
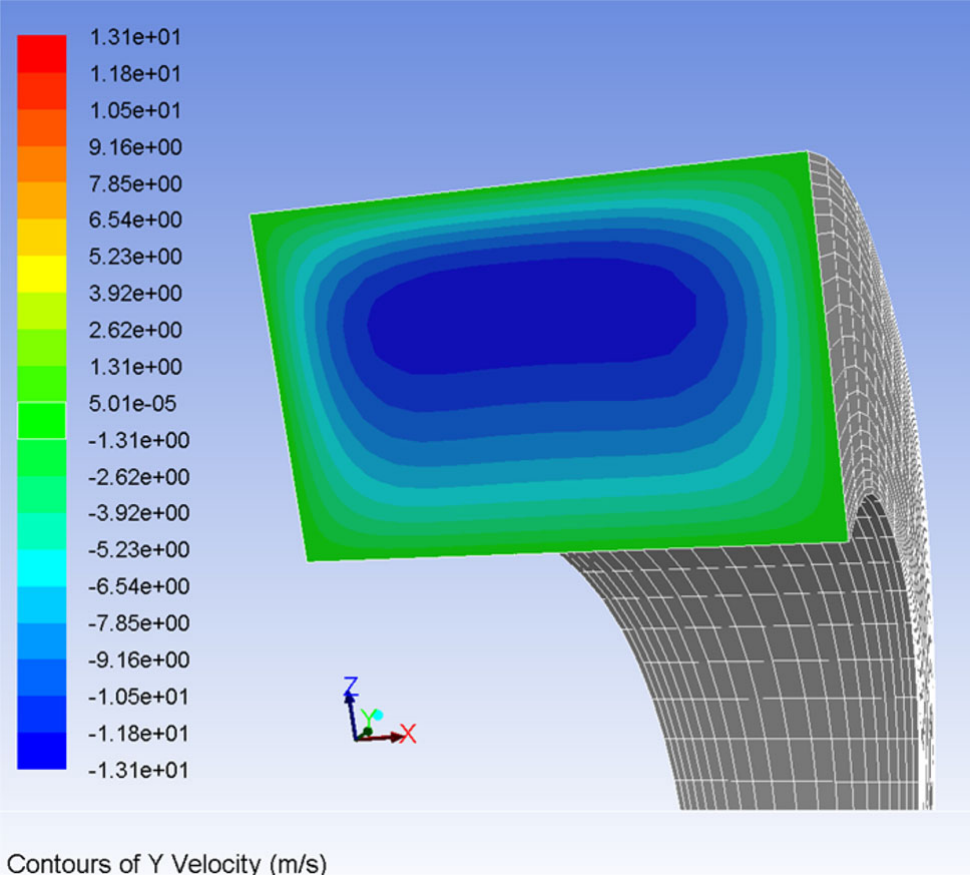
Contours of velocity in an outlet of a helical groove

This allowed a direct comparison of certain parameters described in above formulas, like the velocity or the volume flow rate. It turns out that the flow rate described in (7) and calculated by plugging into variables used in a model—differed from results received from the CFD analysis of a flow through a helical groove. In the model which was formed by 200,000 hexahedral volume elements and had a mesh adjusted such, as to include a boundary layer, the flow was visibly moving off center, towards the external diameter of a helix (see Fig. 21). Because of the need to constantly lubricate the contact area between a slipper and a swash plate, there is a pressure differential between the displacement chamber and the slipper. Since the inlet pressure is varying—the outlet pressure was controlled by the user defined function.

The velocity magnitude given as area weighted average was estimated to be 7.16 m/s, while the volumetric flow rate was 2.89e^−6^ m^3^/s, which is equivalent to 0.173 l/min. That result coincides quite well with hand calculations shown in (11) and (13).

Reynolds number can be calculated by the formula

14 $$Re = \frac{{\rho d_{h} V}}{\mu }$$

where *ρ* (kg/m^3^) is density, *d*_*h*_ (m) is hydraulic diameter, *V* (m/s) is fluid speed and *μ* (kg/ms) is dynamic viscosity.

With *ρ* = 872 kg/m^3^, *V* = 7.19 m/s, *d*_*h*_ = 0.00062 and *μ* = 0.0266 kg/ms,

15 $$Re = \frac{{872\,{\text{kg/m}}^{3} \times 0.0006\,{\text{m}} \times 7.19\,{\text{m/s}}}}{{0.026\,{\text{kg/ms}}}} = 144.7$$

Since *Re* is well below 2300, flow in a gap could be classified as laminar.

#### Leakage in the Gap Between a Piston and a Commutation Sleeve

The second model of leakage had to do with a leakage between a commutation bushing and a piston. There were two configurations taken into account: a concentric position of the piston in the commutation bushing and a skewed arrangement of the piston in the bushing.

##### Concentric Position of a Piston in the Gap

**Fig. 22:**
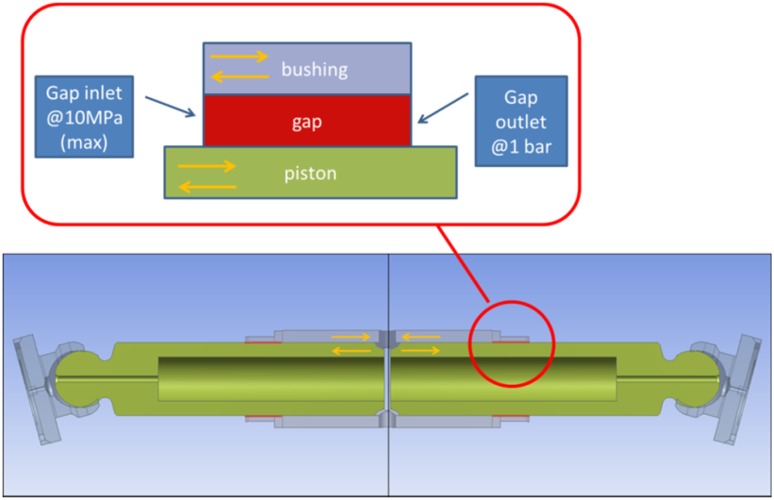
Leak between a commutating bushing and a piston (leak marked in *red*)

The first configuration of the piston, when it is concentric with the commutating bushing, renders the lubrication gap symmetric. The biggest allowed clearance between a piston and a bushing, shown on the manufacturing drawings equals to 24 µm. Therefore, the symmetric gap would have a height of 12 µm. Such gap configuration can be modeled as axisymmetric, which makes the model easier to handle. The lubricating gaps between pistons and a commutating bushing are 11 mm long and are shown in red in Fig. 24. By design both the commutation bushing and two pistons move in a simple harmonic motion: because of a difference in the motion amplitude their velocities differ. The configuration shown in Fig. 22 renders the first out of seven bushing/piston pairs, which are equally spaced around the perimeter of the pump block. Note the yellow arrows which mark the direction of motion of the piston and the bushing—they start their cycle by moving in opposite directions. The piston shown on the right in Fig. 28 will start its work cycle by moving to the right, while the bushing will move to the left.

##### Equations of Motion

**Fig. 23:**
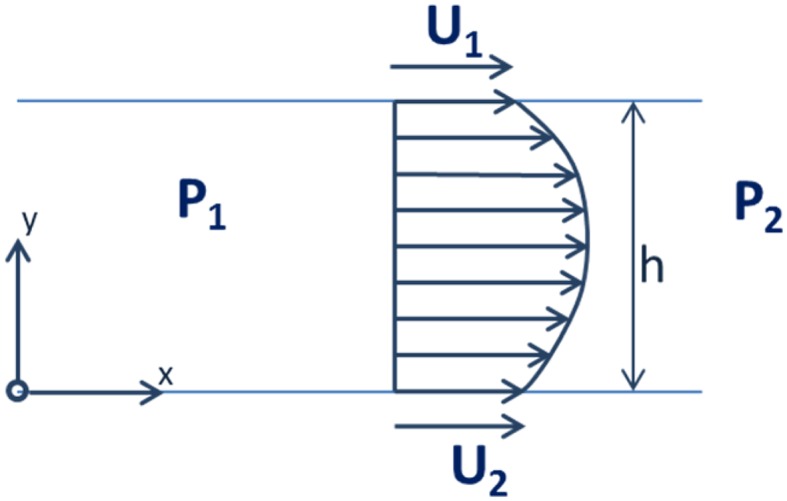
Gap flow between two moving parallel flat plates with an existing pressure gradient

The motion executed by a piston moving in an oscillatory fashion within a commutation bushing effects the flow in a lubrication gap between a piston and a commutation bushing. It could be considered as a particular case of a combined Couette flow and Poiseuille flow, in which there exists a pressure gradient and gap boundary walls are moving (Fig. 23).

16 $$\frac{\partial }{\partial x}\left( {h^{3} \frac{\partial P}{\partial x}} \right) + \frac{\partial }{\partial z}\left( {h^{3} \frac{\partial P}{\partial z}} \right) = 6 \mu \frac{{\partial \left( {U_{2} - U_{1} } \right)h}}{\partial x} + 12 \mu \frac{\partial h}{\partial t}$$

In the case represented by the second model of gap leakage, both plates are moving in the oscillatory motion and they begin the work cycle by moving in opposite directions.

#### Model Description

Assumptions:

- Axisymmetric model of a gap between a commutation bushing and a piston.
- Density controlled by user defined function (dependent on pressure).
- Moving walls (velocity of each wall assigned a velocity of either element).
- Input pressure controlled by user defined function.
- Output pressure at the atmospheric level.

Axisymmetric model of the leakage gap was based on the assumption that both piston and bushing are concentric. That simplification applies to some situations and makes calculations easier. The model consisted of 48,000 quad elements that spanned the gap of 12 µm height and 11 mm width, bounded by two moving walls: the top one belonging to a bushing and the bottom one belonging to a piston. Refer to Fig. 22, which describes the topology of the gap formed by the commutating bushing and one of the pistons.

**Fig. 24:**
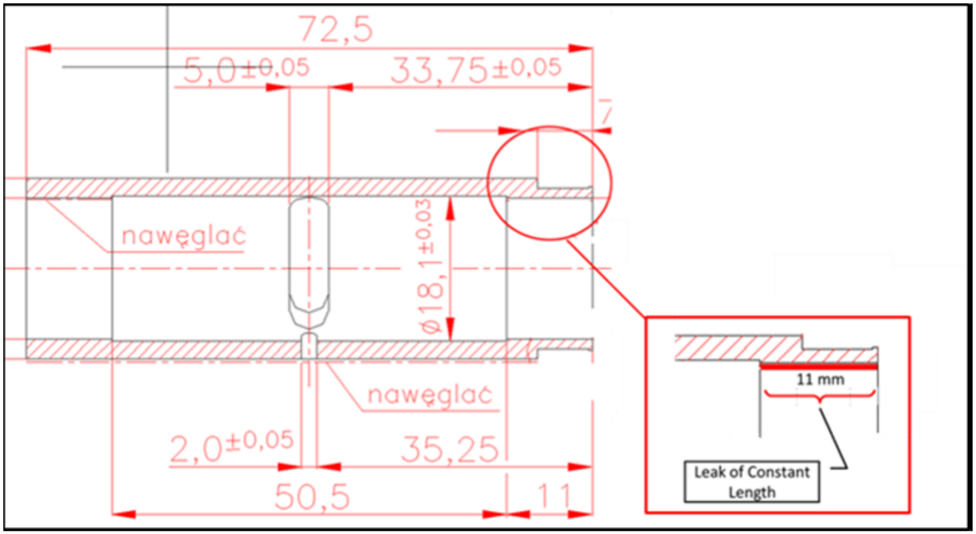
Topology of the gap length in model 2—between the commutation bushing and the piston

In model 2, the gap between a commutating bushing and the piston is constant owing to the topology of the bushing, and is equal to 11 mm (Fig. 24).

In order to verify the correctness of the model, a verification check was conducted to compare the analytical results with the outcome of the simulation. Although in model 2 both bounding walls were moving, thus creating a flow in which a combination of pressure gradient and moving walls were influencing the behavior of fluid, in the verification model only the pressure gradient was considered, since it lends itself to a simple check with the Poiseuille Eq. (17), which assumes that there is no relative motion between a piston and a bushing and only the pressure gradient exists so that

17 $$Q = \frac{{\Delta p \pi d h^{3} }}{12 \mu l}$$

where *Q*—volumetric flow rate (m^3^/s); Δ*p*—pressure gradient (Pa); *d*—piston diameter (m); *h*—gap width (m); *μ*—dynamic viscosity (kg/ms); *l—*length of the gap (m).

By plugging in Δ*p* = 9.89 MPa, *d* = 0.018 m, *h* = 12e^−6^ m, *μ* = 0.026 kg/ms, *l* = 0.011 m one gets the flow rate equal to 2.82e^−7^ m^3^/s or 1.69e^−2^ l/min. That is exactly the results that could be obtained from the simulation of model 2 (an axisymmetric model of a lubrication gap between the commutation bushing and the piston), with stationary walls.

If the influence of moving parts (like a cylinder or a bushing) is included, then (17) is transformed into

18 $$Q = \pi d \left( {\frac{{\Delta ph^{3} }}{12 \mu l} \pm v_{0} \frac{h}{2}} \right)$$

where *ν*_0_ is the velocity of the piston and the sign depends on the direction of the flow.

**Fig. 25:**
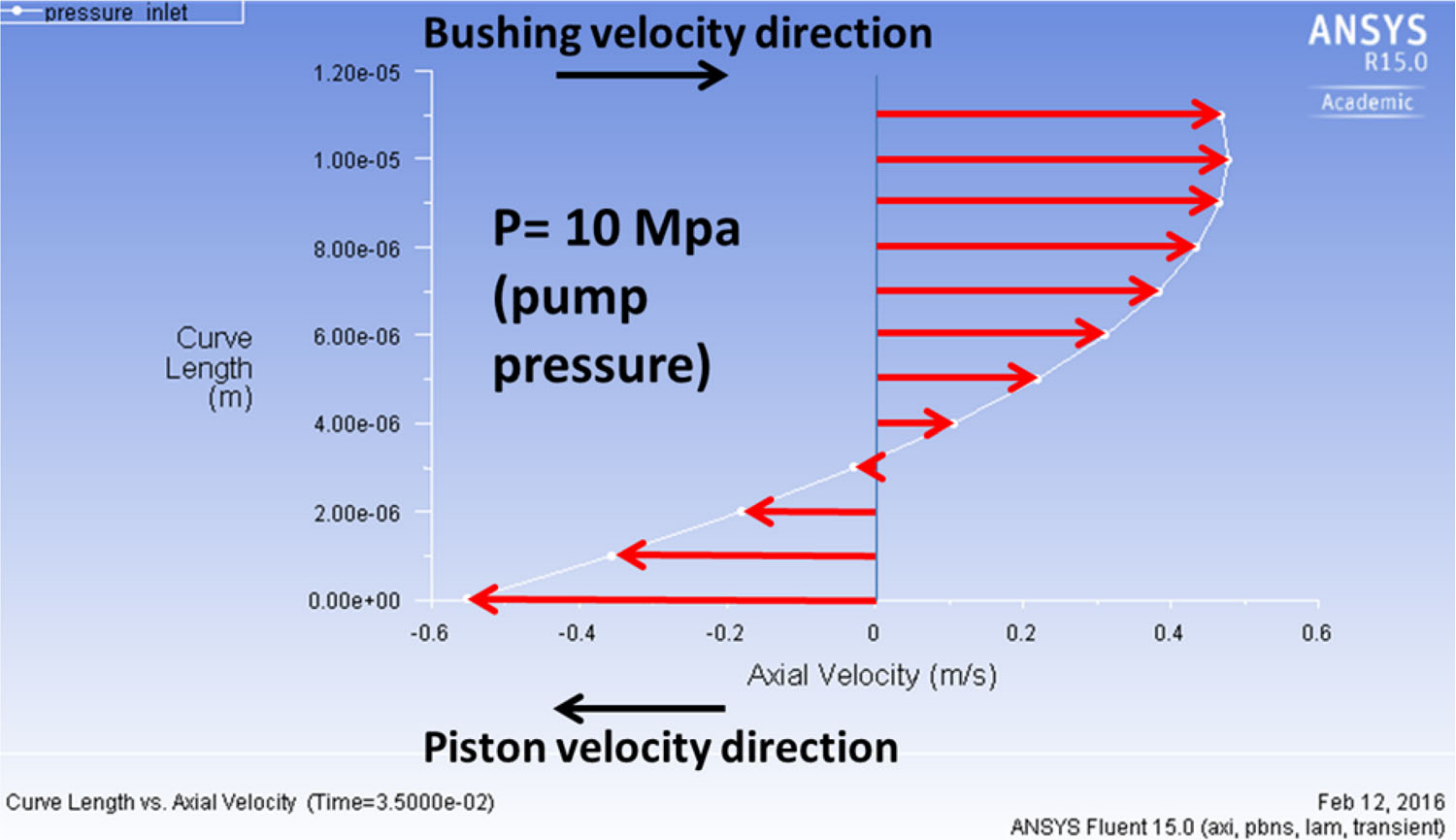
Flow velocity at the gap inlet (time step 0.035 s, 1000 rpm, 100 % capacity)

In Fluent a harmonic motion of both moving elements can be specified in a user defined function. The plot of the velocities of the right piston and the bushing is shown in Fig. 25. The pump was operating with 100 % capacity at 1000 rpm. The inlet of the gap was exposed to the pressure in the displacement chamber [changing from 0.2 (inlet port pressure) to 10 MPa (outlet port pressure)], while the outlet of the gap was kept at the atmospheric pressure.

The flow of fluid in the gap was effected by the motion of both walls. The following plot (Fig. 25) shows the vector plot of fluid velocity field at the time step of 0.035 s, clearly affected by the wall movement. The bushing (upper wall) moves to the right, while the piston (lower wall) moves to left, bringing some of the fluid back to the pump, overcoming the pressure gradient of 10 MPa/11 mm).

**Fig. 26:**
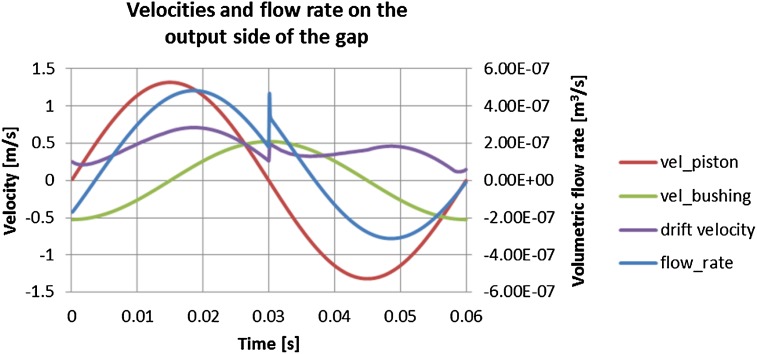
Volumetric flow rate in a gap formed by a moving piston and a moving bushing

**Fig. 27:**
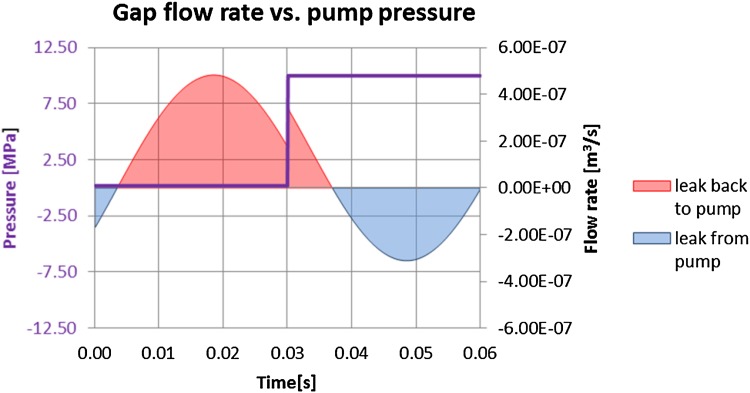
Impact of pump pressure on flow rate

There are several combinations of how the velocities of the piston and the bushing may interact, since during the work cycle they either move in the same or the opposite direction. Because of the strong influence of friction and the resulting drift velocity, fluid in the gap is driven back to the displacement chamber in spite of the fact that in the second part of the work cycle there is high pressure of 10 MPa applied at the gap inlet side. The influence of velocities imposed by the bushing-piston pair on the gap flow can be clearly seen in Figs. 26 and 27.

In Fig. 27 that phenomenon is shown in the form of red-shaded area which represents the leak of oil moving back to the pump in spite of the fact that fluid is exposed to high pressure in the displacement chamber. The pressure gradient over the gap length of 11 mm is 10 MPa. Therefore, when calculating the net flow rate out of the gap one should consider the whole work cycle, since the influence of moving parts on the fluid should be taken into account, as well as the symmetry of that configuration. The following results were obtained from the numerical analysis: the volumetric flow rate in the gap between a concentrically located piston and a bushing equal to 1.47e^−7^ m^3^/s or 8.81e^−3^ l/min.

The type of flow can be determined by calculating the Reynolds number as

19 $$Re = \frac{{\rho \times V_{inlet} \times d_{h} }}{\mu }$$

where *ρ*—density (kg/m^3^); *V*_*inlet*_—inlet velocity (m/s); *d*_*h*_—hydraulic diameter = 2 (r_bushing_ – r_piston_) (m); *μ*—dynamic viscosity (kg/ms);

20 $$Re = \frac{{872\,{\text{kg/m}}^{3} \times 9.4\,{\text{m/s}} \times 2 \times 12\,{\text{e}}^{ - 6} \,{\text{m}}}}{{0.026\,{\text{kg/ms}}}} = 7.6$$

The flow in the gap between a moving piston and a moving bushing is laminar.

##### Skewed Position of a Piston in the Gap

**Fig. 28:**
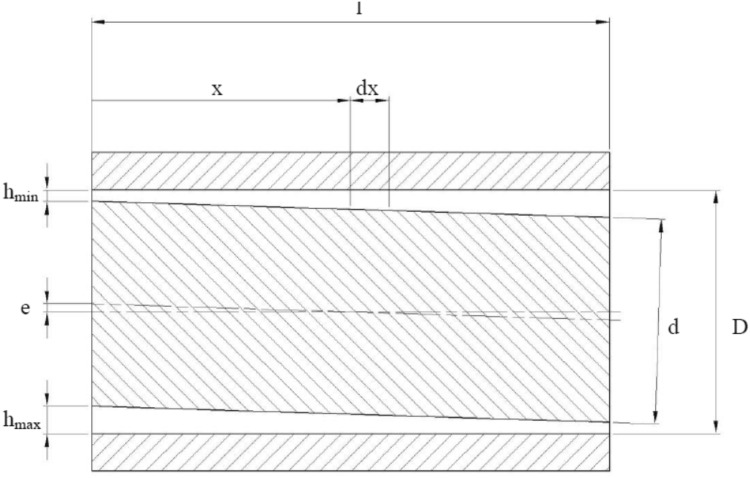
Skewed position of a piston in a commutation bushing

The second leakage model described in the previous section can be modified by introducing a slant to a position of a piston with respect to a commutation bushing. That kind of a piston orientation could be caused by a radially acting force. In Fig. 28 a cross section through a piston slanted in a commutation bushing is shown.

The assumption was that the gap height will be reduced by 90 %.

#### Equations of Motion

The motion of fluid between two surfaces, one of which is stationary, can be described with a Reynolds equation

21 $$\frac{\partial }{\partial x}\left( {h^{3} \frac{\partial P}{\partial x}} \right) + \frac{\partial }{\partial z}\left( {h^{3} \frac{\partial P}{\partial z}} \right) = 6\mu \frac{{\partial \left( {U_{2} - U_{1} } \right)h}}{\partial x} + 12\mu \frac{\partial h}{\partial t}$$

where *h*—gap height (m); *U*_*1*_ *, U*_*2*_—velocities at boundaries with respect to *x* (m); *P*—pressure (Pa); *ρ*—density (kg/m^3^); *μ*—dynamic viscosity (kg/ms).

The terms on the left hand side of the equation represent flow due to pressure gradients across the domain, while the terms of the right hand side represent flows induced by motions of the bounding surfaces and shear-induced flow by the sliding velocities of the boundaries.

The gap has a height which is a function of the position and could be written as:

22 $${\text{h}}\left( {\text{x}} \right) = h_{mim} + \frac{{\left( {x - x_{0} } \right)^{2} }}{2R}\quad {\text{for}}\quad 0 < x < L$$

##### Model Description

Assumptions:

- Model of a gap with a piston skewed in a bushing.
- Heat input (oil temperature at 49 °C).
- Viscosity controlled by user defined function (dependent on temperature).
- Density controlled by user defined function (dependent on pressure).
- Moving walls (velocity of each wall assigned a velocity of either element).
- Input pressure corresponding to pressure in the displacement chamber.
- Output pressure at the atmospheric level.

**Fig. 29:**
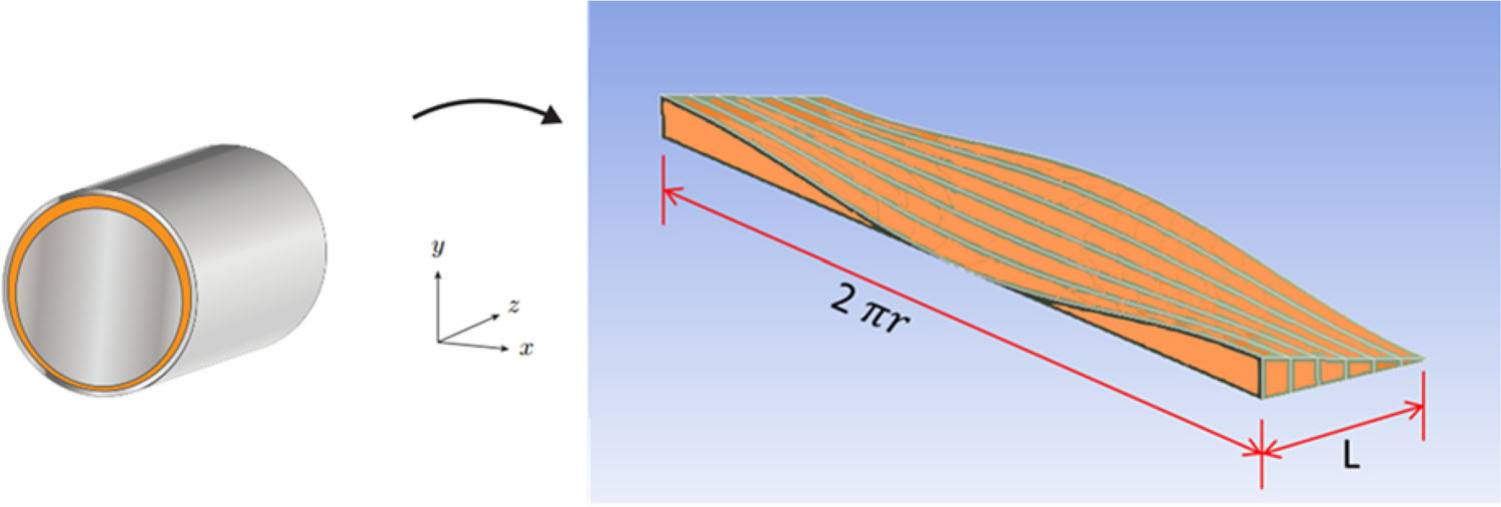
CAD model of the development of a lubricating gap which is formed by a skewed piston

The lubricating gap created by a slanted orientation of a piston in a bushing can’t be modeled with axial symmetry. A different method has to be implemented in order to capture physics of that phenomenon. A three dimensional model of a gap, whose dimensions would change in every of the three directions. Can be represented by the development of a curved surface (Fig. 29).

The perimeter of a piston of radius “*r*” forms the width of the lubrication gap (2 · *π* · *r*) in Fig. 29, the height of the gap is defined as a radial distance between the piston and the bushing, whereas the gap’s length corresponds to a length of a piston engaged in the bushing. Because of a large disproportion between two of the gap dimensions (length and width are at the level of mm, while its height is defined in µm)—a CAD model is very hard to handle owing to that scale difference of sizes. In order to create elements whose aspect ratio would be acceptable, one would have to build a model containing close to 20 M volume elements. Such models are not easily handled by ordinary desktop computers, therefore a different approach was taken by the authors.

A finite difference method was used and the results were calculated in Excel. The program had an iterative solver turned on and a matrix of points depicting a surface developed from a gap formed by a skewed piston was formed.

**Fig. 30:**
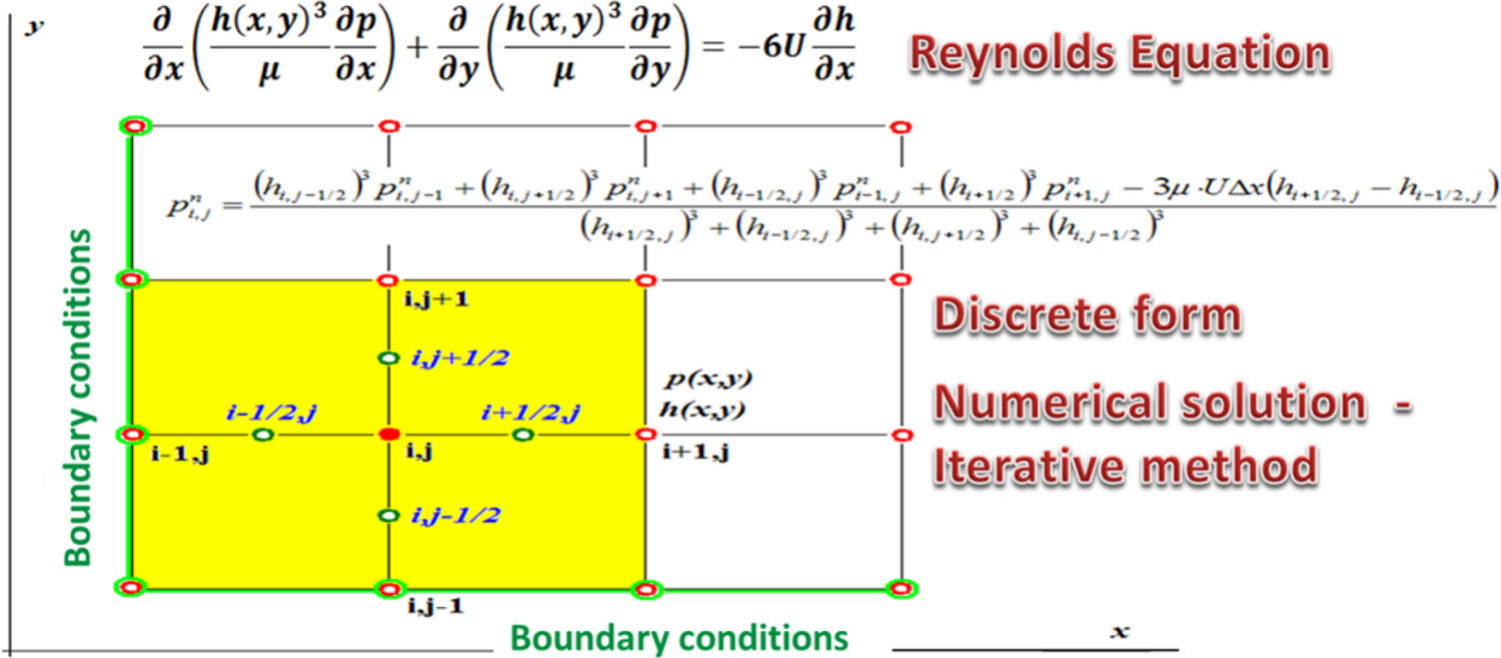
FDM Stencil to calculate Reynolds equation

Each point on a grid was assigned either a boundary condition (if it was located on the edge of the gap) or was given a formula shown in Fig. 30, which was a transposed form of Reynolds equation.

**Fig. 31:**
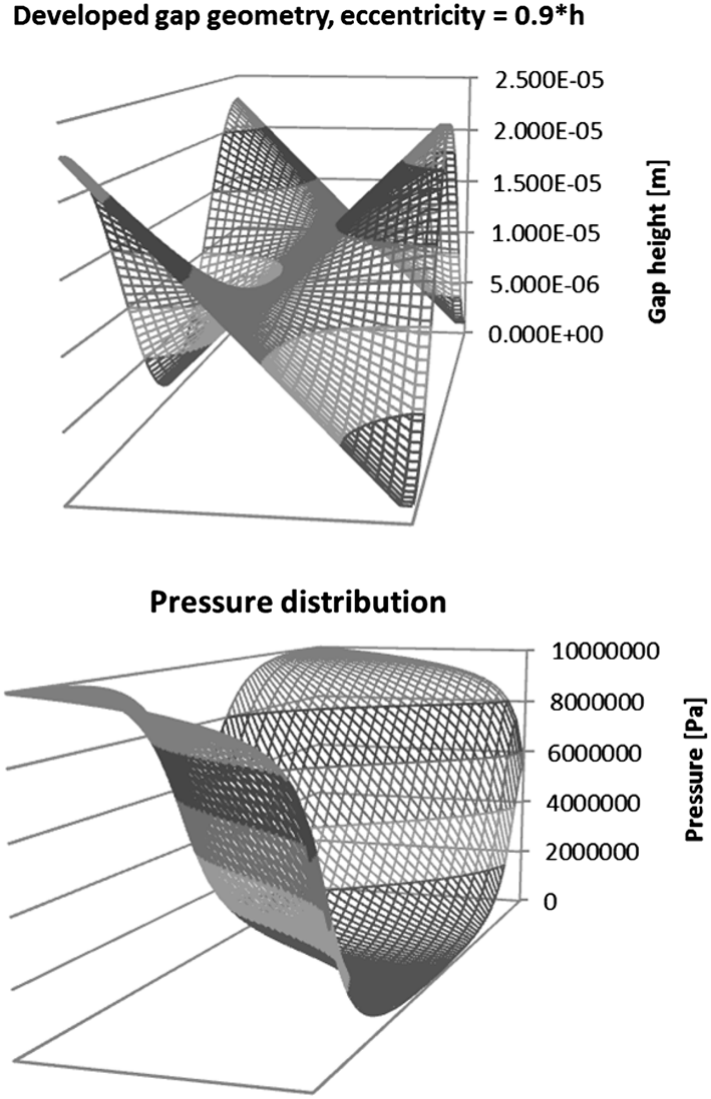
Geometric development of the gap (*top*) and pressure distribution at time = 0.04 s (*bottom*)

The radial clearance between the bushing and the piston was 12 µm and the length of the gap was 11 mm. When the gap geometry was developed into a 3D solid—its width *b* (equal to 2 · *π* · *r*) was 56.5 mm (assuming a piston diameter of 9 mm). A grid covering the length and the width of the gap was created with steps of 0.67 mm along the width and 0.42 mm along the length. The example of pressure distribution is shown in Fig. 31, where the piston is skewed in the bushing (it was assumed that the eccentricity of the piston with respect to the bushing was 0.9 · *h*, where *h* is the height of the gap. The plots show the transient pressure at 0.04 s through the 0.06 s work cycle. At that point the bushing was moving with the velocity of 0.26 m/s in the direction of the pressure gradient (Δ*p* = 9.8 MPa), while the piston had velocity of 1.14 m/s, moving in the opposite direction. Since the iterative calculations result in the distribution of pressure a formula for velocity was used to arrive at the flow rate through the gap. In the formula for velocity

23 $$v = \frac{1}{2 \cdot \mu } \cdot \frac{\partial p}{\partial x} \cdot z^{2} + c_{1} \cdot z + c_{2}$$

boundary conditions were imposed: for the height of the gap, *z* = 0, velocity of the flow equal to that of the piston, i.e. *u*_2_ and for z = h, the velocity of the flow was equivalent to the velocity of the bushing *u*_1_, giving

24 $$v = \frac{1}{2 \cdot \mu } \cdot \frac{\partial p}{\partial x} \cdot \left( {z^{2} - z \cdot h} \right) + \frac{z}{h} \cdot \left( {u_{1} - u_{2} } \right) + u_{2}$$

Since

25 $$Q = b \cdot \mathop \int \limits_{0}^{h} v dz$$

therefore

26 $$Q = \frac{b}{12 \cdot \mu } \cdot \frac{\partial p}{\partial x} \cdot h^{3} + b \cdot \frac{h}{2} \cdot \left( {u_{1} - u_{2} } \right) + u_{2} \cdot h$$

The flow rate through the gap which was formed by a slanted piston with eccentricity equal to 0.9 · *h* was calculated to be 5.11e^−6^ m^3^/s or 5.11e^−3^ l/min.

Since we were dealing with a transient problem (both the piston and the bushing forming a gap were moving) the pressure distribution in a gap was calculated for 60 steps with a step of 0.001 s.

#### Leakage in the Gap Between a Commutation Sleeve and Pressure Ports

**Fig. 32:**
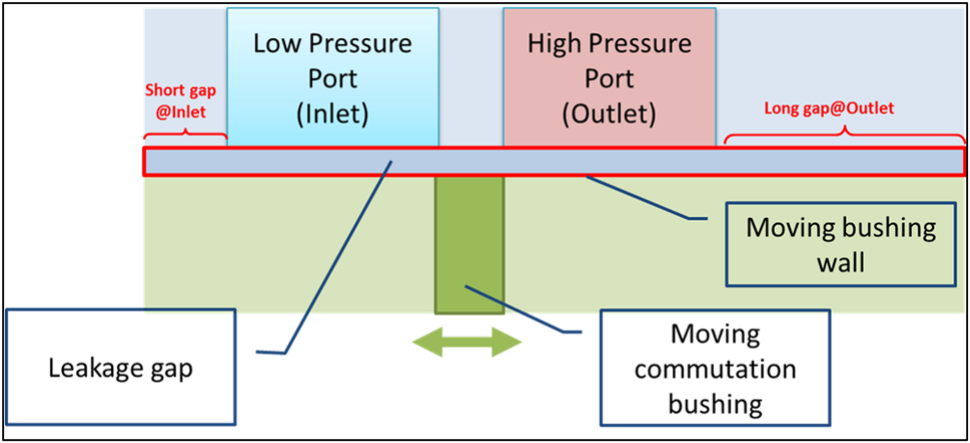
Leakage between a commutation bushing and pressure ports

The third leak that is to be considered (no. 3 in Fig. 13) occurs in the gap between the pressure ports and the commutation bushing. It was approached with an assumption that only the simplest configuration is to be analyzed, namely the one of the piston concentric with the bushing. Such a model lends itself to be treated as an axisymmetric problem. The schematic describing the model is shown in Fig. 32.

The gap connects the pressure ports with each other, but also each of the ports with the atmosphere. The initial configuration depicted in Fig. 32 shows the long gap connecting the high pressure port (outlet) with the atmosphere. The short gap connects the low pressure port to the ambient pressure.

##### Governing Equations

The third leakage model of the PWK pump is similar to model no. 2 except that previously two boundary walls were moving, while in the current model only the commutation bushing is subjected to motion. The stationary wall is formed by the pump wall with pressure ports, and the moving wall corresponds to the moving plate in the Couette flow description.

The generalized Reynolds equation that describes the conservation principle between two parallel plates can be stated as

27 $$\frac{\partial }{\partial t }\left( {\rho h} \right) + \frac{1}{2}\frac{\partial }{\partial x}\left( {\rho hU} \right) = \frac{\partial }{\partial x}\left( {\frac{{\rho h^{3} }}{12\mu }\frac{\partial P}{\partial x}} \right) + \frac{\partial }{\partial y}\left( {\frac{{\rho h^{3} }}{12\mu }\frac{\partial P}{\partial y}} \right)$$

where *h*—gap height; *μ*—fluid viscosity; *U*—velocity at boundary (with respect to *x*); *P*—pressure; *x*—is in the direction of the gap; *y*—is in the perpendicular direction to *x*.

The terms on the right hand side of Reynolds equation represent the flow due to pressure gradients. The left hand side shows the flows induced by normal (squeeze) motions of the bounding surface and the shear induced flow by the surface sliding with velocity U.

##### Description of the Model

Assumptions:

- Axisymmetric model of a gap between a commutation bushing and pressure port.^1^
- Height 12 µm, length of the gap equal to 48.1 mm.
- Heat input (oil temperature at 49 °C).
- Viscosity controlled by user defined function (dependent on temperature).
- Density controlled by user defined function (dependent on pressure).
- Dynamically moving mesh.
- Input pressure controlled by user defined function.
- Output pressure at the atmospheric level.

**Fig. 33:**
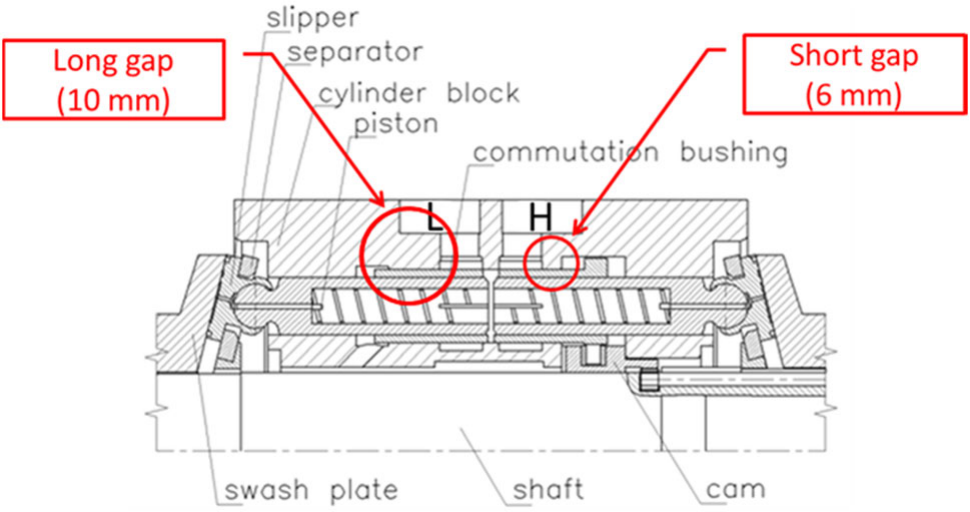
Location of leakage between a commutation bushing and pressure ports

The model consisting of a gap and peripheral structures like the pump inlet, pump outlet and the commutation bushing was discretized with 92,000 quadrilateral elements, out of which 54,000 elements defined the gap proper, which was 12 µm high and 48.1 mm long. The location of lubrication gaps described below is shown in Fig. 33.

It portrays an asymmetry between the leakage gaps circled in red. That configuration is arbitrary and dependent on the judgment of the designer, who wants to optimize the pump performance. Both gaps, whose lengths are 6 and 10 mm, respectively connect the pump inlet and outlet to atmosphere, thus creating a direct path for fluid loss.

An analysis was performed to find out how the intuitive guess could be quantified. The reasoning was that in order to minimize the fluid loss, a long gap should be located on the high pressure side of the pump because of a smaller pressure gradient in the gap and thus a smaller flow rate. In the model both pressure ports and a lubrication gap were stationary, while the bushing was assigned a harmonic motion. The model of the bushing consisted of the fluid release port and solid walls—their velocity was controlled by an user defined function (UDF).

**Fig. 34:**
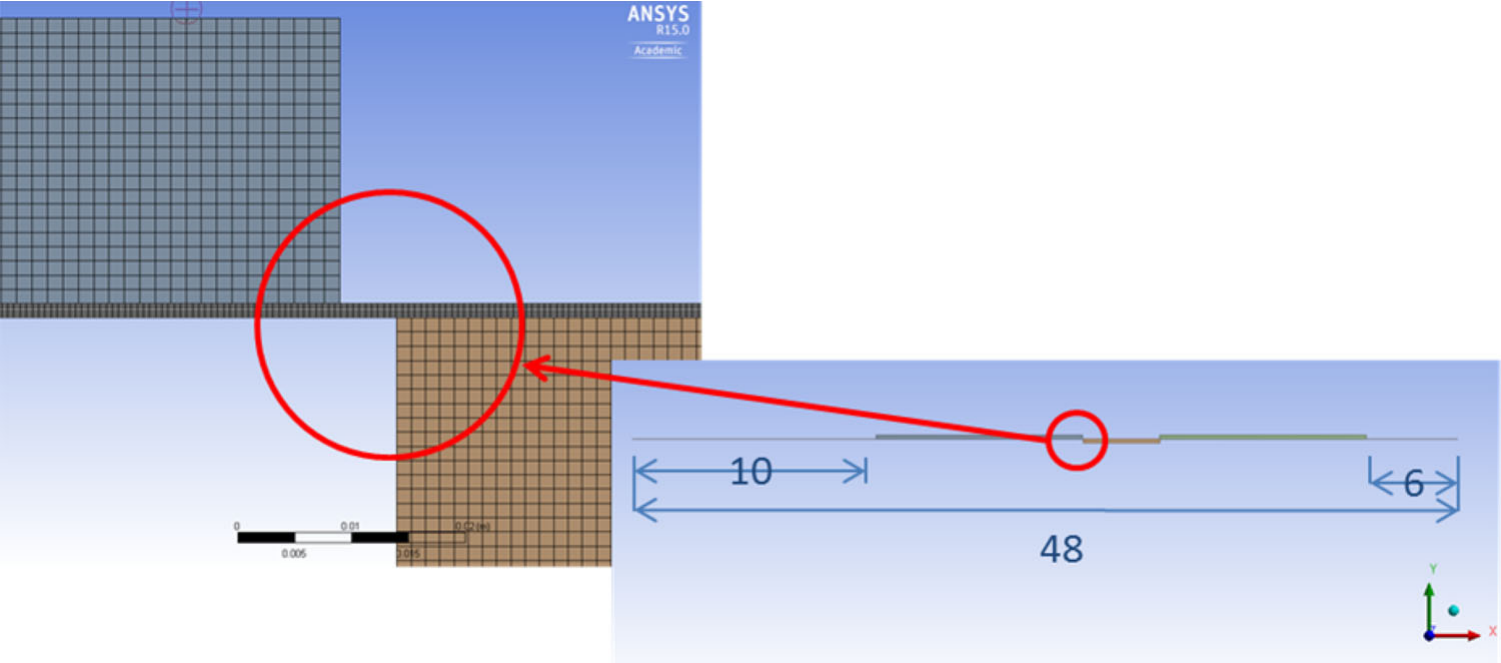
General topology of the axisymmetric model (*right*) and details showing a commutation bushing, pump inlet and the gap (*left*)

The fluid was heated to 49 °C and dynamic viscosity was controlled by UDF. With the shaft rotation of 1000 rpm the working cycle lasts 0.06 s. (In PWK pumps the capacity can be set with the stepper motor, by adjusting the commutation bushing position with respect to the location of both pistons and pressure ports). In the analyzed case corresponding to 100 % pump displacement, the initial location of the port of the commutation bushing was set under the bridge which separates high and low pressure ports (Fig. 34).

**Fig. 35:**
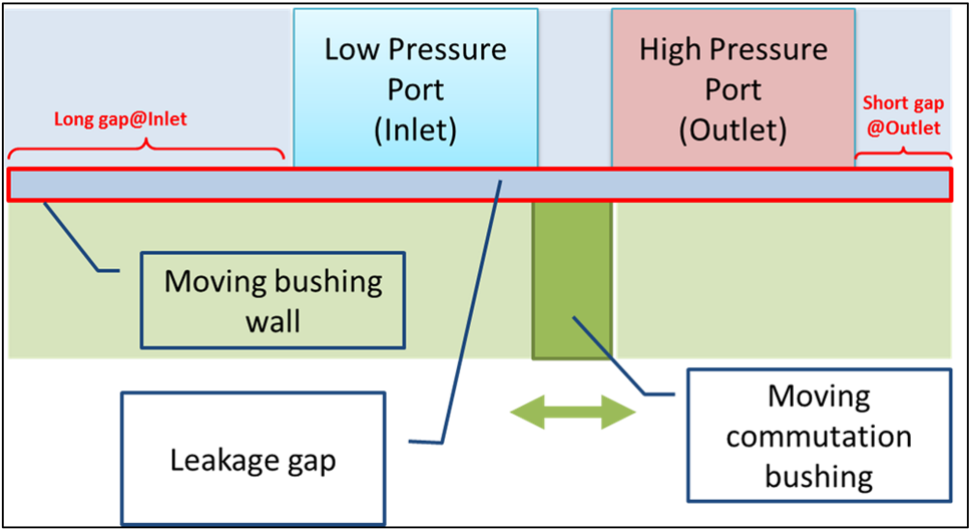
Leakage between a commutation bushing and pressure ports (“Inverted” configuration)

The long lubricating gap was positioned on the side of the high pressure port (see Fig. 32). Subsequently the topology was inverted (see Fig. 35) and the long gap was placed next to the low pressure port, so the influence of gap topology on the fluid flow rate could be checked.

**Fig. 36:**
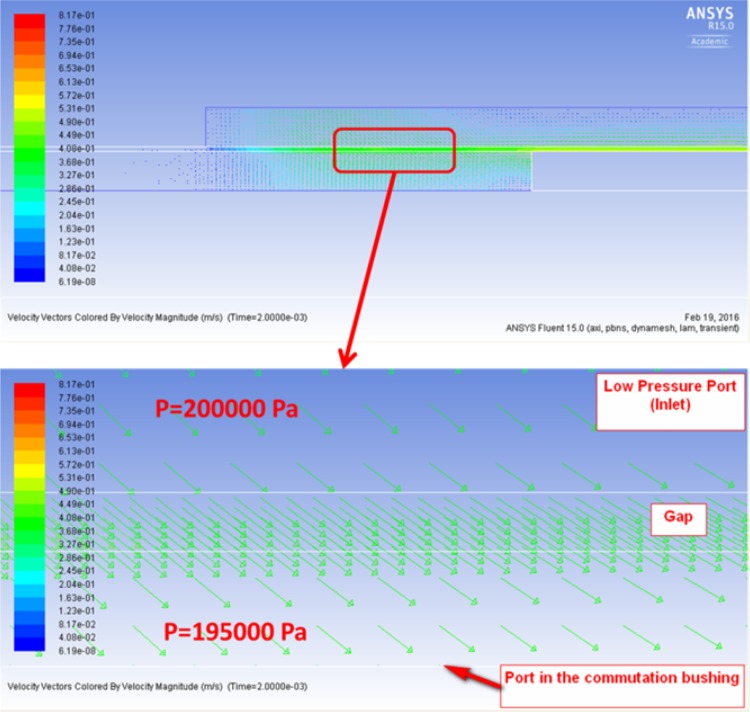
Flow of fluid in the suction part of the working cycle

In order to force the fluid motion between high/low pressure ports and the commutation bushing, additional pressure adjustments had to be carried out. In order to fill the displacement chamber through the port in the bushing wall, the flow from the low pressure port (inlet) where pressure was set at 200,000 Pa, had to move into lower pressure volume. Therefore the pressure in the displacement chamber was adjusted according to the boundary condition adopted from the global model of the pump (195,000 Pa, cf. Fig. 36).

Similarly, to enable the fluid flow from the displacement chamber into the high pressure port, an excess pressure had to be assigned to the bushing port in the middle of the working cycle, when the pistons started to compress the fluid. Then the pressure in the displacement chamber was increased by 15,000 Pa with respect to the pressure value in the high pressure port, whose value was set to 10 MPa.

**Fig. 37:**
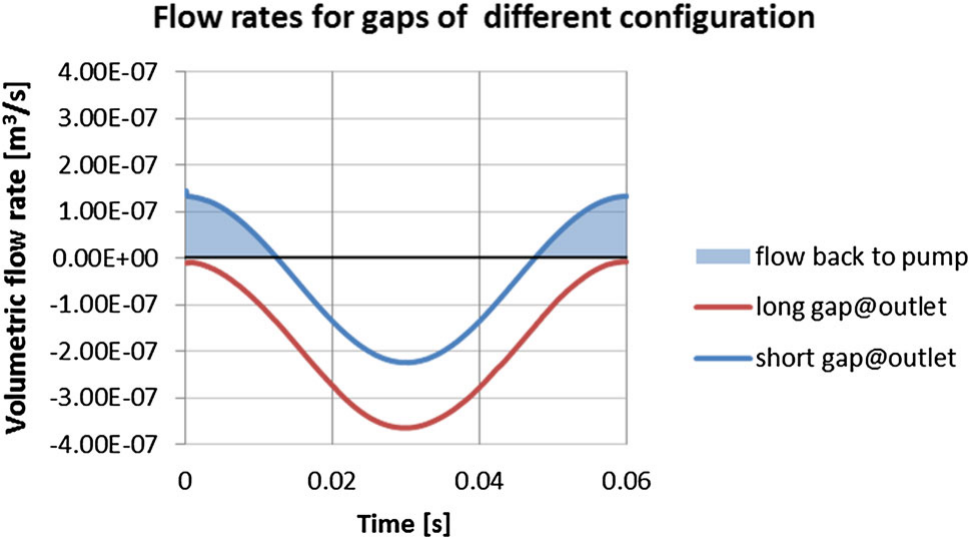
Flow rate in gaps connecting a high pressure port with the atmosphere

Consequently, the plots showing the comparison of flow rates in gaps of different configurations were prepared. One remark has to be made at this point: an axial piston pump is a positive displacement pump and as such produces a constant flow at a given speed of a drive shaft. The presented plots consider a flow rate produced by a single piston. The flow of the whole pump assembly consists of a superposition of its components, which ultimately gives a constant flow. Figure 37 shows volumetric gap flow rate dependent on the length of the gap connecting high pressure port with the atmosphere.

The plot points to a counterintuitive solution: a flow rate through a shorter gap on the high-pressure side (represented by a blue curve in Fig. 37) is smaller than the one through a longer gap. It looks like the bigger pressure gradient which occurs in the case of a shorter gap is not a sufficient factor to drive more fluid out of the pump). The major factor is played by the drift velocity, which influences the flow rate through the short gap connecting the high pressure port (outlet) with the atmosphere.

**Fig. 38:**
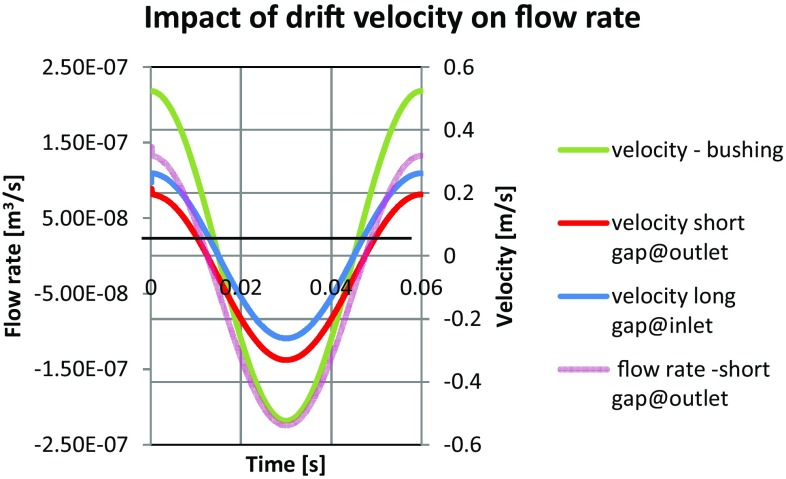
Drift velocity impact of flow rate through the gap near the high pressure port

The plot in Fig. 38 shows velocities of the bushing and the flow through the gap, measured at both ends of the gap. There is a close correspondence between the velocity of the bushing (colored in green) and the volumetric flow rate (colored in pink). By integrating the area under the curve in Fig. 38 one can get the following quantification: the flow rate from the high pressure port through the short gap is 2.76e^−3^ l/min and through the long port it is 1.12e^−2^ l/min. At the inlet port side of the pump. The flow rate through the longer gap was: 2.03e^−5^ l/min, while the flow through the short gap was: 1.14e^−4^ l/min. The summary of the leakage is shown in Table 4.

**Table 4:** Comparison of flow rate values for different lengths of the lubrication gaps between pressure ports and a commutation bushing

Flow rate from inlet port through		Flow rate from outlet port through		Total flow rate (l/min)
Short gap	1.14e^−4^ (l/min)	Long gap	1.12e^−2^ (l/min)	1.13e^−2^
Long gap	2.03e^−5^ (l/min)	Short gap	2.76e^−3^ (l/min)	2.76e^−3^

The total flow rate through the lubrication gap between pressure ports and the commutation bushing clearly points to the fact that when a shorter gap is placed near the high pressure port, the flow rate is four times smaller. That is depicted in Table 4.

Taking the bigger value of the Reynolds number for either gap one gets:

28 $$Re = \frac{{\rho \times V_{inlet} \times D}}{\mu } = \frac{{872\,{\text{kg/m}}^{3} \times \,0.33\,{\text{m/s}} \times 0.023\,{\text{m}}}}{{0.044 \,{\text{kg/ms}}}} = 150$$

That stipulates the flow in the gap between pressure ports and the commutation bushing is laminar.

**Fig. 39:**
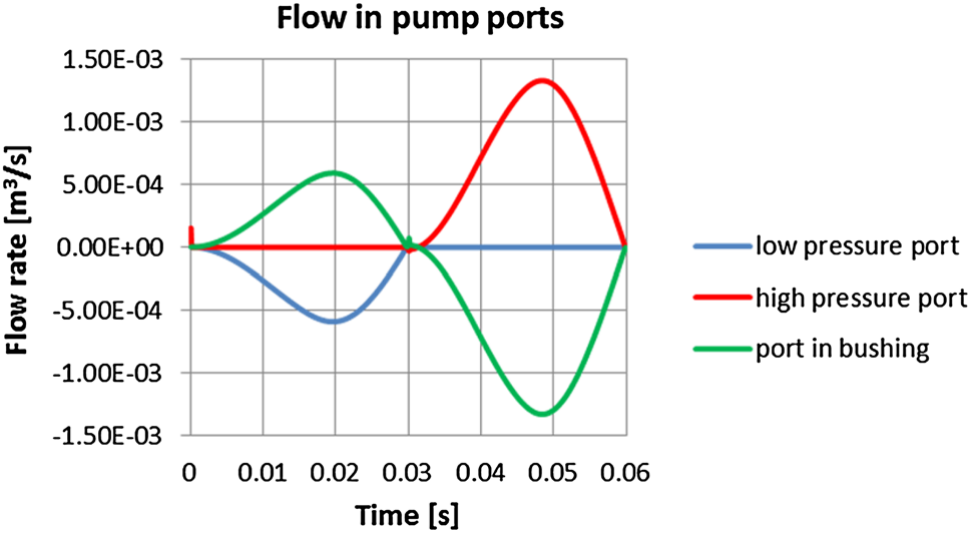
Flow between high and low pressure ports and the relief port in the bushing

There is one more gap leak in model 3 discussed above, that should be given consideration. That concerns a possible leak from a high pressure port to the low pressure port. The analysis results confirm that flow from both high and low ports is mostly exchanged with the flow from the bushing port. Therefore in the suction phase all fluid is pumped from the low pressure port through the bushing to the displacement chamber and in the compression phase the fluid is transported to the outlet port (see Fig. 39). There is no leakage from the high pressure port to the low pressure port (the curve representing the flow to the low pressure port is flat in the interval between 0.03 and 0.06 s).

Among factors playing a crucial role in the behavior of hydraulic flow in a lubrication gap is fluid viscosity. Its dependence on temperature was shown in Fig. 19. There was a study conducted on the sensitivity of volumetric flow rate to the increase of oil temperature. Intuitively the flow of fluid should increase when the viscosity lowers, because flow velocity is inversely proportional to the dynamic viscosity. And the flow rate as the product of velocity and the cross section area should follow the velocity. It turns out that such claim is not true in case when the drift velocity comes into effect. Model of a leakage between pressure ports and the commutation bushing served as an example. Analysis considered two configurations of topology (lubrication gaps of different lengths that connected pressure ports to atmosphere were alternately switched (in the first run the longer gap connected the high pressure port to the atmosphere, and in the second run the shorter gap replaced the longer one). Each run was repeated with the energy option switched on. That corresponded to applying temperature of 49 °C to the fluid. Such condition effected the flow rate as seen in figures below.

**Fig. 40:**
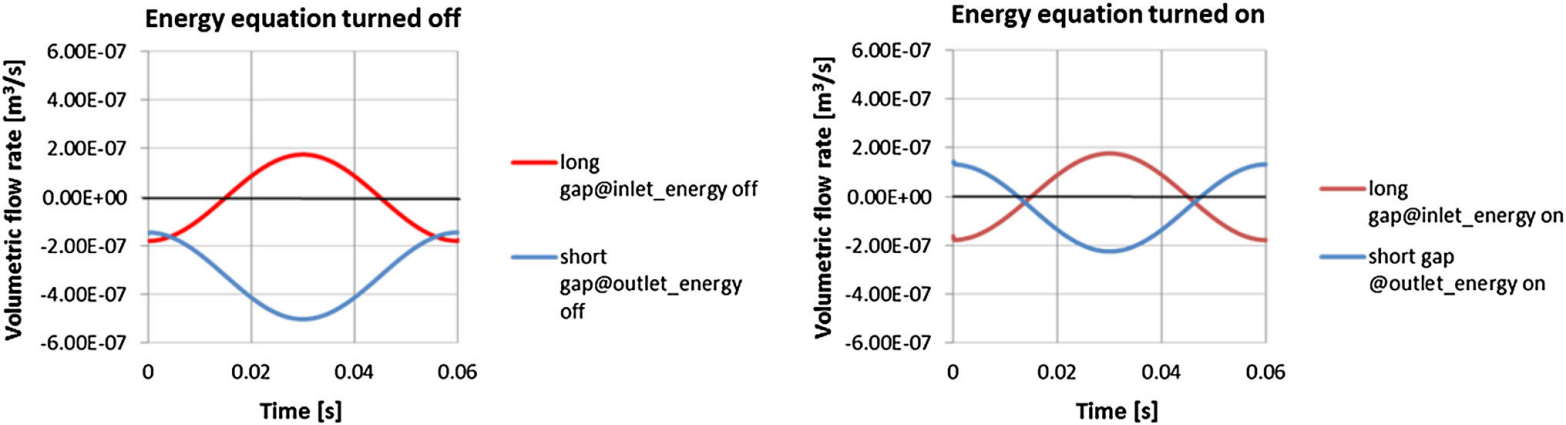
Comparison of volumetric flow rates with different thermal conditions

Figure 40 depicts the effect of temperature of the fluid on the flow rate when the high pressure port is connected to atmosphere with a short gap. With the energy equation on and the oil temperature set to 49 °C (the right plot in Fig. 40) the flow from the high pressure port slows down, compared to the flow rate shown on the plot with no energy equation switched on (left plot in Fig. 40). The total volumetric flow rate corresponding to the flow with no heat flow considered is: 1.95e^−2^ l/min, while the much lower total flow rate corresponding to the simulation with the energy equation turned on is equal to: 2.74e^−3^ l/min which is seven times smaller. The reason for that is in the fact that the temperature rise decreases the viscidity of fluid which makes less susceptible to the pressure gradient and more responsive to the influence of a moving bushing. Table 5 summarizes the results of comparison of the flow rate in gaps of different lengths with no energy equation turned on.

**Table 5:** Comparison of flow rates in lubricating gaps with the energy equation turn off

Flow rate from inlet port through		Flow rate from outlet port through		Total flow rate (l/min)
Short gap	1.39e^−4^ (l/min)	Long gap	1.12e^−2^ (l/min)	1.18e^−2^
Long gap	1.14e^−4^ (l/min)	Short gap	1.94e^*−*2^ (l/min)	1.95e^−2^

**Fig. 41:**
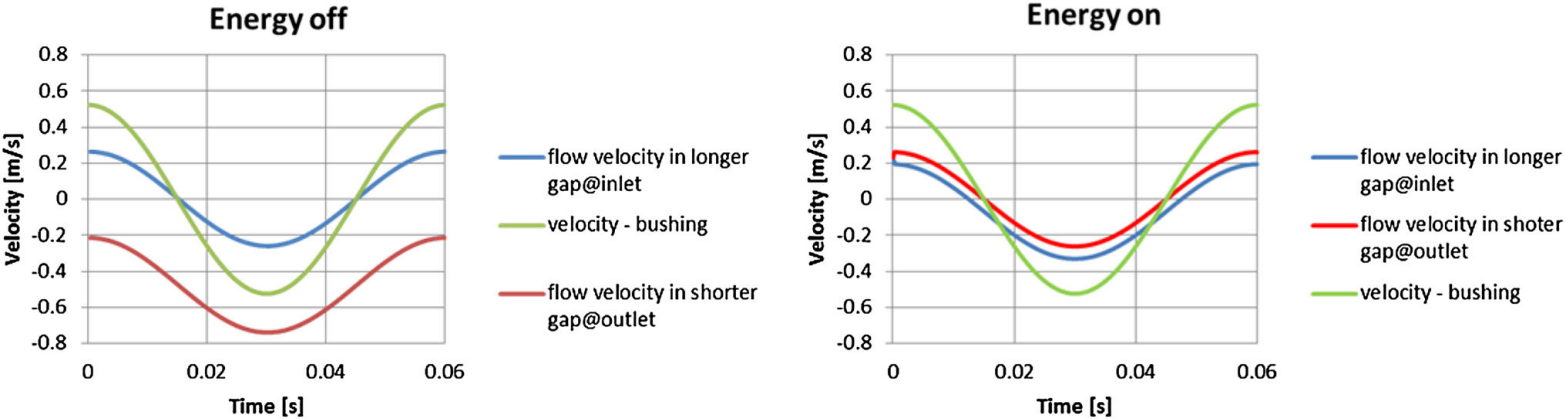
Comparison of flow velocities in a long gap connected to high pressure port and different thermal conditions

With the energy equation turned off the ratio in total leakage between gaps of different lengths is 1.7. When the energy equation is turned on, that ratio increases to 7. Finally the velocity profile of flows with and without the energy equation switched on is presented in Fig. 41.

The effect of temperature on oil viscosity and thus the flow rate can be visible clearly in the configuration of the shorter gap connecting high pressure port with the atmosphere. The flow velocity in a gap near the high pressure port shown in red on the left plot in Fig. 41 has negative direction during the whole cycle, which means that the pressure gradient overcomes the influence imposed by the motion of the bushing. The drift velocity impacts the flow from the high pressure port gap when the energy equation is turned on. Then the movement of fluid in the gap follows the motion of the bushing throughout the cycle (right plot in Fig. 41).

### Summary of Leakage Flow Calculations

Table 6 below summarizes volumetric flow rate in all three analyzed models. Models 2 and 3 are axisymmetric.

**Table 6:** Balance of leakage through the lubrication gaps in an axial piston pump

Model no.	Volumetric flow rate per cylinder (l^3^/min)	Volumetric flow rate per pump (l^3^/min)	Percentage of leakage (%)
Model 1 (leakage in the helical gland)	1.73e^−1^	2.4	92.23
Model 2 (leakage between the commutation bushing and the piston)	8.81e^−3^	1.23e^−1^	4.73
Model 3 (leakage between pressure ports and the commutation bushing)	1.13e^−2^	7.91e^−2^	3.04

## Conclusions

Applying CFD simulation to a PWK axial piston pump design process shows that it could be useful in situations when analytical solution is complicated or unavailable. Additionally, the analysis of flow in gaps of different configurations revealed the practical advantage of applying a numerical analysis. For obvious reasons a new design of the axial pump has to be rely on virtual prototyping and that is particularly important when dynamic phenomena are considered and the transient simulation has to be performed. Often coefficients describing fluid flow through the analytical formulas are inaccurate and only owing to numerical simulation precise assessment of the flow parameters can be obtained. Numerical simulation of flow in lubrication gaps poses severe challenges closely linked with the spatial scale of the problem, as well as with the complexity of the physical phenomenon. It is quite easy to model one aspect of the piston-cylinder configuration, namely the case—when both objects are concentric. Then the axisymmetric model of the lubrication gap can be applied. The situation gets complicated when lateral loading on the piston is to be taken into effect and a piston is forced to assume an eccentric or skewed position with respect to a cylinder. That opens way to the solution of the Reynold’s equation by iterative methods.

In order to simulate the full coupled phenomenon of flow in a lubrication gap more effort would have to be expended than described in this paper. And such analysis would have to include fluid–structure interaction and heat transfer. Such task is doable specially with the use of approach presented above—when multiscaling is taken into account and a global problem could be transferred into a local one.

## Footnotes

1 A skewed model of the gap based of a Finite Difference stencil could be constructed in a manner similar to one shown in Sect. 2.7.6.1. Pressure at the commutation bushing window could be imposed as a transient boundary condition changing with each step of the working cycle.
